# Palaeoproteomic identification of the original binder and modern contaminants in distemper paints from Uvdal stave church, Norway

**DOI:** 10.1038/s41598-024-63455-4

**Published:** 2024-06-04

**Authors:** Zahra Haghighi, Meaghan Mackie, Anne Apalnes Ørnhøi, Abigail Ramsøe, Tone Marie Olstad, Simon James Armitage, Christopher Stuart Henshilwood, Enrico Cappellini

**Affiliations:** 1 SFF Centre for Early Sapiens Behaviour (SapienCE), University of Bergen, Bergen, Norway; 2 Globe Institute, University of Copenhagen, Copenhagen, Denmark; 3 School of Archaeology, University College Dublin, Dublin, Ireland; 4 ArchaeoBiomics, Department of Life Sciences and Systems Biology, University of Turin, Turin, Italy; 5 The Norwegian Institute for Cultural Heritage Research (NIKU), Oslo, Norway; 6 Department of Geography, Royal Holloway University of London, Egham, Surrey UK; 7 Evolutionary Studies Institute, University of the Witwatersrand, Johannesburg, South Africa

**Keywords:** Mass spectrometry, Proteomics, Archaeology

## Abstract

Two distemper paint samples taken from decorative boards in Uvdal stave church, Norway, were analysed using palaeoproteomics, with an aim of identifying their binder and possible contaminants. The results point at the use of calfskin to produce hide glue as the original paint binder, and are consistent with the instructions of binder production and resource allocation in the historical records of Norway. Although we did not observe any evidence of prior restoration treatments using protein-based materials, we found abundant traces of human saliva proteins, as well as a few oats and barley peptides, likely deposited together on the boards during their discovery in the 1970s. This work illustrates the need to fully consider contamination sources in palaeoproteomics and to inform those working with such objects about the potential for their contamination.

## Introduction

Characterisation and identification of the materials used to produce cultural and historical artefacts is one of the main aims of researchers in the field of art conservation and restoration. Determining the materials used to produce artwork and their chemical composition is very important for deciding the best conservation practices for a particular object. In addition, the information obtained from the characterisation of the materials is not only used to make inferences about the production of the object and its degradation state, but also to attempt the attribution of the object to a specific artist or time period. Occasionally this information may also be used to differentiate fakes and forgeries from originals^1^.

Studying the organic compounds found within such objects is an analytically challenging task as these materials have undergone deterioration processes and are most likely to be present within a complex matrix of inorganic materials. Nevertheless, analytical developments and new techniques introduced to this field have played a crucial role in tackling such challenges^2,3^. In studying ancient proteins, their biological source, and their degree of degradation, palaeoproteomics has already shown promising and accurate results^4^ due to the development of highly sensitive mass spectrometry-based workflows. It has been used to elucidate the protein content of historical and archaeological artworks^5^, artefacts^6^, and biological remains^7–9^, even from samples over one million years old^10^.

Here we present the results of palaeoproteomic analyses of two distemper paint samples from Uvdal stave church (Uvdal stavkirke), dating back to 1170 A.D, in Norway. Today, there are only 28 mediaeval stave churches remaining in Norway, 17 of which decorated with distemper paintings, representing an important part of Norwegian history and building heritage^11^.

In the distemper technique, pigments are mixed with animal or plant glue as the binding medium, in contrast with tempera painting which usually uses egg yolk. During the decoration of stave churches, the distemper paint was most likely applied warm and dried fast, leaving little time for the painter to adjust and correct or to use a wet-in-wet technique. Distemper decorative paintings characteristically have a porous surface and matte finish and are vulnerable to moisture and other changes in the indoor climate^12^. The use of distemper may reflect the painter's appreciation for the surface texture and visual appeal of the paint. However, despite oil painting being an established technique at this time, the decision to use distemper to paint stave churches was likely driven by practical considerations. During this period, the required ingredients for making distemper paintings were more affordable and attainable than those for oil paintings. In terms of the binder itself, linseed oil was costly compared to the animal-based glues. Moreover, linseed oil had to be ordered from the larger cities, which increased costs due to transportation and added logistical complications when ordering sufficiently large amounts for such extended projects. It might, therefore, have been easier to order animal skins and make the glue on-site when needed. Additionally, distemper is more cost-effective when using certain pigments, such as using chalk instead of lead white to produce white paint. Also importantly, distemper paint dries quickly, making it possible to work continuously, whilst oil paint instead needs weeks to dry. Therefore, distemper can be a preferable technique for large-scale paintings such as those in Uvdal stave church^13^. In the case of Uvdal, most of the surfaces inside the church are painted, which was not unusual for decoration of stave churches.

**Figure 1:**
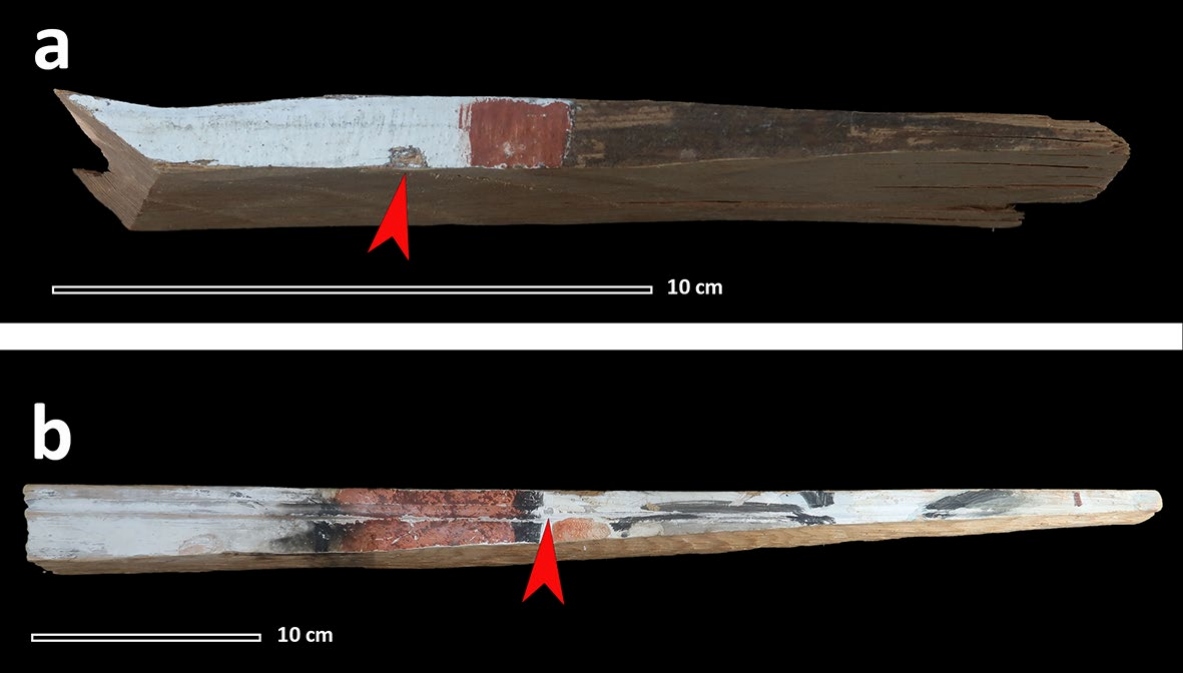
Samples taken from UV1 (**a**) and UV2 (**b**). The sampling point for each painted board is indicated by the red arrow. The dimensions (height × width × depth cm) of the sampled painted boards are: (5.4 × 18 × 1.2 cm) and (2–4 × 50 × 1.2 cm) respectively. Copyright: Norwegian Institute for Cultural Heritage Research (NIKU), Oslo.

In the 1650s, several of the stave churches in the valley of Numedalen, including Uvdal stave church, were decorated with distemper paint. Due to the proximity of the churches, the relatively brief timeframe in which the entire project was completed, and the shared characteristics in these paintings, it is most likely that the same painter and painting technique were used to produce all these works. The initial phase commenced at Gol stave church in Hallingdal in 1652, followed by Rollag stave church^14^ as well as the Numendal stave churches in Nore and Uvdal stave church in 1655 and 1656, respectively^15^. Furthermore, in 1684, Uvdal stave church was altered and repainted, and in 1723 extended with transepts. The samples analysed in this study were taken from painted boards (Fig. 1) discovered under these transepts during a major restoration undertaken in 1978. We therefore believe that the painted boards date to either 1656 or 1684^16^. After discovery, these boards were taken to the Directorate for Cultural Heritage, and afterwards to the Norwegian Institute for Cultural Heritage Research (NIKU) for storage. No additional information is available about these painted boards or about the team who has discovered them. In the early 1990s, almost all the paint decorations of Uvdal stave church underwent consolidation treatment using sturgeon glue^17^. The analysed boards were transferred to NIKU before this event and there is no record of them having undergone any subsequent conservation or consolidation treatment^12,18^. They, therefore, represent ideal candidates for the study of the original composition of the distemper binder.

Previously published analyses using multiple different techniques such as spot testing, immuno-fluorescence microscopy (IFM), enzyme-linked immunosorbent-assay (ELISA), and fourier transform infrared attenuated total reflectance spectroscopy (FTIR-ATR) on a number of samples from several stave churches, including that at Uvdal, suggest the presence of collagen proteins, and specifically sturgeon glue^19^. Nevertheless, sturgeon glue is unlikely to be the original binder in this case, as it easily chips off when applied in layers^20^ and importing sturgeon would be prohibitively expensive for a work of this size. Therefore, we assume that it was introduced as a conservation measure. Using sturgeon glue as a consolidation treatment for distemper paints was developed by the Directorate for Cultural Heritage and the Norwegian Institute for Cultural Heritage Research around 30 years ago^17,21^ and has been a common practice ever since^22^. However, in using the methods mentioned above, it has not been possible to confidently characterise the original paint binder, highlighting the necessity for employing a different technique, such as palaeoproteomics, that could provide accurate and detailed information regarding the original binder within the distemper paints. Confident identification of the original binder would not only guide future conservation efforts, but also offer valuable insights about the cultural practices and resource allocation at the time of production, especially considering that there is little written evidence to draw upon.

The main objective of our study was to identify the biological tissue and the species of origin of the proteinaceous binding media initially used in the Uvdal stave church paintings. Due to the untargeted nature of the proteomic approach, we also examined other proteinaceous traces in our samples, possibly deriving from undocumented restoration or conservation treatments.

## Methods

### Sample preparation

Two distemper samples were mechanically removed from white areas of the decorated boards using a scalpel under a microscope at NIKU. Since distemper paint decorations typically feature a base layer of white or greyish-white throughout the design, we aimed to sample from these areas. The rationale for this approach is that, while all the painted areas in distemper paintings contain the same binder, the base layer is not subject to interference from subsequent applications of paint, making the task of sampling and later experiments easier.

The methodology for sample preparation followed the procedure published by Mackie et al.^23^ and all chemicals used are LC–MS grade. Approximately 1 mg of each specimen was removed and transferred to Eppendorf Protein Lo-Bind tubes and incubated at 80 °C for 2 h in 100 μL 2 M guanidinium hydrochloride solution (GdnHCl; Sigma-Aldrich, Germany) with 10 mM Tris(2-carboxyethyl)phosphine hydrochloride (TCEP; Sigma-Aldrich, Germany) and 20 mM chloroacetamide (CAA; Sigma-Aldrich, Germany), buffered with 100 mM Tris (Invitrogen, Germany). A sample preparation blank was processed alongside the samples. Protein digestion was performed under agitation at 37 °C in-solution, first with 0.2 μg rLysC (Promega, Sweden) for 2 h before being diluted to a final concentration of 0.6 M GdnHCl using 25 mM Tris in 10% acetonitrile (ACN, Thermo Fisher Scientific, Germany) in water and digested with 0.8 μg Trypsin (Promega, Sweden) overnight. The supernatant and sample pellet were both digested at the same time. Samples were then acidified to pH 2 using 10% trifluoroacetic acid (TFA; Merck, Germany) in order to quench digestion. Peptides were collected and desalted on in-house made C18 extraction StageTips as described previously^8^. Samples were stored at −18 °C until mass spectrometry (MS) analysis could be performed.

## Nano-liquid chromatography–tandem mass spectrometry (nLC-MS/MS)

Samples were eluted from the StageTips into a 96 well MS plate using 30 μL 40% ACN, 0.1% formic acid (FA; Sigma-Aldrich, Germany) in LC–MS grade water (18.2 MΩ·cm resistivity). Samples were placed in a vacuum centrifuge at 40 ºC until approximately 3 μL of solution was left and then rehydrated with 8 μL of 0.1% TFA, 5% ACN. Samples were then separated on a 15 cm column (75 μm inner diameter) in-house laser pulled and packed with 1.9 μm C18 beads (Dr. Maisch, Germany) on an EASY-nLC 1200 (Proxeon, Denmark) connected to an Exploris 480 Orbitrap mass spectrometer (Thermo Fisher Scientific, Germany) on a 77 min gradient. Buffer A was milliQ water. The peptides were separated with increasing buffer B (80% ACN and 0.1% FA), going from 5 to 30% in 50 min, 30% to 45% in 10 min, 45% to 80% in 2 min, held at 80% for 5 min before dropping back down to 5% in 5 min and held for 5 min. Flow rate was 250 nL/min. The column temperature was maintained at 40 °C using an integrated column oven. A wash-blank method using 0.1% TFA, 5% ACN was run in between each sample to minimise cross contamination.

The Exploris 480 was operated in data dependent top 10 mode. Spray voltage was 2 kV, S-lens RF level at 40, and heated capillary at 275 °C. Full scan mass spectra were recorded at a resolution of 120,000 at *m/z* 200 over the *m/z* range 350–1400 with an automatic gain control (ACG) target of 300 and a maximum injection time of 25 ms. HCD-generated product ions were recorded with a maximum ion injection time set to 118 ms and an ACG target of 200, recorded at a resolution of 60,000. Normalised collision energy was set at 30% and the isolation window was 1.2 *m/z* with the dynamic exclusion set to 20 s.

### Data analysis

The resulting raw files were analysed using the MaxQuant software version 1.6.3.4^24^. Several runs of the software in an iterative manner were performed against different datasets (Swissprot, then human and bovine proteomes from Uniprot, and finally a database containing the hits from the previous searches). Parameters common amongst all runs are as follows: tolerances were those preset for Orbitraps, using a semi-tryptic search with up to two missed cleavages. Minimum peptide length was set to 7. Carbamidomethylation was set as a fixed modification, while methionine oxidation, deamidation of asparagine and glutamine, hydroxylation of proline, and glutamine and glutamic acid to pyroglutamate were set as variable modifications. Protein and peptide identifications were filtered by a false discovery rate (FDR) of 0.01 and manually filtered by at least 2 different non overlapping peptides. Contaminant proteins were assessed using the contamination.fasta provided by MaxQuant which includes common laboratory contaminants^25^. These protein hits were excluded from further analysis, except when relevant to our analysis.

To confirm the species identification, all identified peptides underwent BLASTp searches^26^ with the NCBI database to validate species identifications and to rule out conserved peptides between species, as well as manual spectrum inspection using MaxQuant viewer.

### Calculation of deamidation

DeamiDATE 1.0^27^ was employed to calculate deamidation rate of the samples. The evidence and peptide files from MQ were used as the input. DeamiDATE calculates the site-specific deamidation of proteins present within a sample in addition to bulk deamidation. The site-specific approach differentiates between deamidation that occurs due to rapid events (deamidation that occurs during protein extraction in the laboratory) and high half-times deamidation events. In theory, the latter should only occur over longer time spans and be observed in degraded samples. This feature helps to distinguish genuine ancient proteins from modern contaminants.

## Results

### Binding media identification

Several proteins were confidently identified in each sample, with a summary provided in Table 1. Multiple species are represented in each sample, indicating a complex mixture of proteins. Details of all the proteins identified, as well as their species-specificity, can be found in Supplementary Tables S1, S2, S3, S4.

**Table1:** Summary of the identified proteins from each of the two distemper samples studied.

Museum ID	LC/MS sample ID	Species ID^a^	No. of proteins	No. of identified razor peptides (semi-tryptic)	No. of MS2 spectra
UV1	2009	*Bos taurus*^b^(9); *Homo sapiens* (32); *Avena sativa* (1); *Hordeum vulgare*/*Triticum aestivum* (1)	72	2480	4695
UV2	2010	*Bos taurus*^b^ (8); *Homo sapiens* (6)	23	781	1125

^a^The number of proteins specific to each identified taxon is indicated in brackets. The other proteins counted in the No. of Proteins column are not species-specific.

^b^Although the same set of observed protein fragments can also be assigned with equal probability to other bovinae species which have never been endemic to Norway, these species can be excluded due to the sample’s context.

Findings regarding the original binder proteins demonstrated the presence of cow (*Bos taurus*) collagen proteins–namely collagen 1 alpha 1 (COL1A1), collagen 1 alpha 2 (COL1A2) and collagen 3 alpha 1 (COL3A1) in both samples (Supplementary Fig. S1, S2, S3, S10, S11, S12). These proteins can be found in several tissues but are especially indicative of connective tissues such as bone, tendon, and skin. Bovine specific collagen peptides were also recovered from both samples, indicating its use as the paint binder. No collagen peptides that specifically correspond to sturgeon or any other fish were recovered.

**Figure 2:**
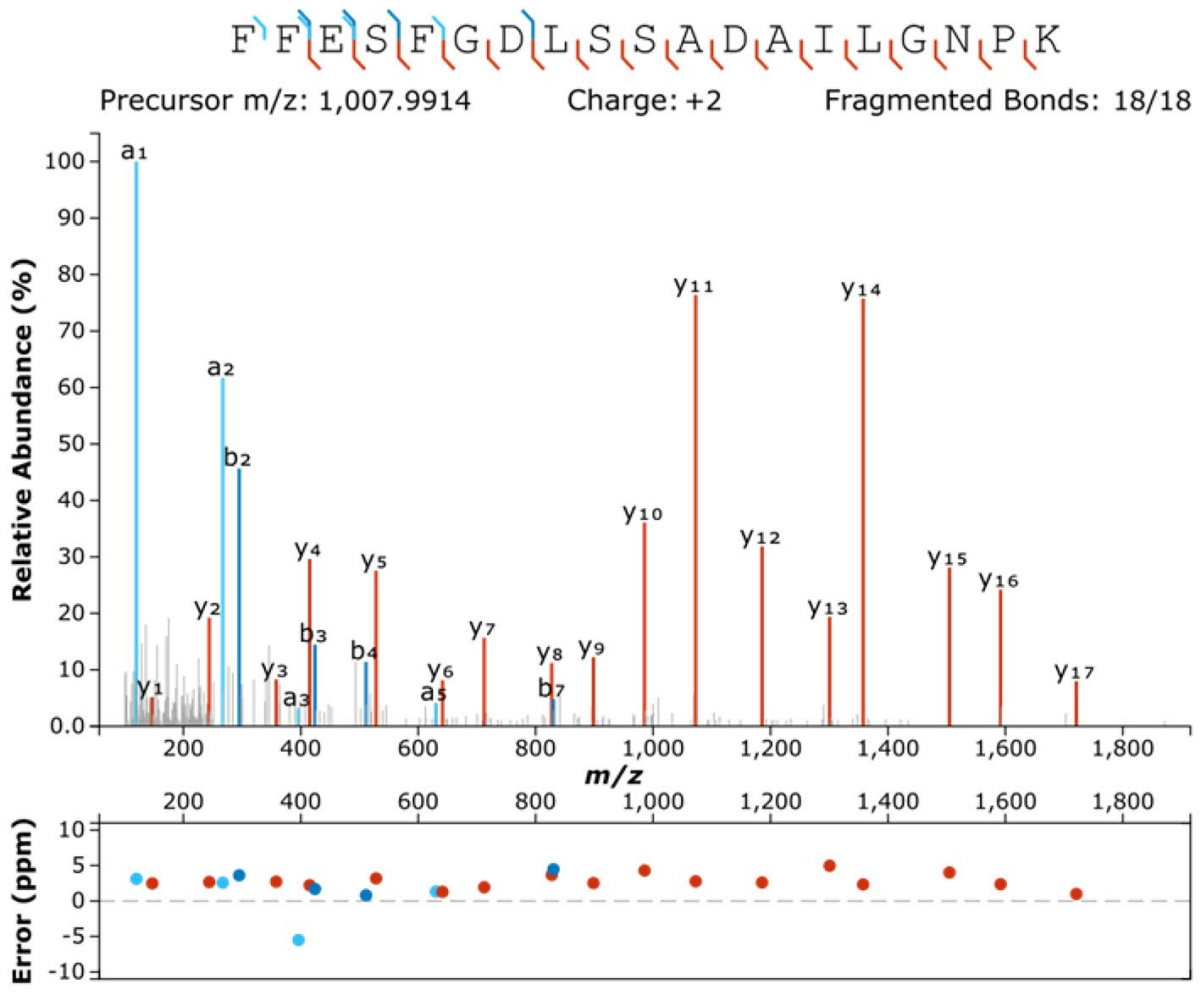
The MS/MS spectra of doubly charged FFESFGDLSSADAILGNPK peptide (m/z 1,007.9914) recovered in UV2, which is specific to bovine haemoglobin foetal subunit beta protein. Detected b- and y- ions are highlighted in blue and red, respectively, with corresponding mass errors in ppm below. Spectrum visualised using Interactive Peptide Spectral Annotator^29^.

In addition to *Bos taurus* collagen proteins, we were able to find both bovinae keratin and bovinae haemoglobin foetal subunit beta (HBBF, Fig. 2, Supplementary Fig. S9). The latter only exists in calves up to a few months old^28^.

### Contaminants

In both samples, in particular sample UV1, we found a considerable number of proteins appearing to derive from human saliva, which were not present in the control blank extraction. Out of 72 proteins in UV1, more than 50 of them have been found in modern dried human saliva^30^. We verified our assignment by finding *Homo sapiens* or Hominidae specific peptides for several of these proteins, including alpha-amylase, Mucin-5B, and Lactotransferrin (Supplementary Tables S2, S4). Overall, good quality spectra allowed for confident protein and species assignments (Supplementary Figs. S5, S6, S14, S15). Additionally, one protein from *Avena sativa* (oats) and one from *Hordeum vulgare* (barley) were detected. Specifically, we were able to verify the assignment of 12S seed storage globulin 1 protein corresponding to *Avena sativa* and Gamma-hordein-1 to *Hordeum vulgare* by specific peptides and good quality spectra (Supplementary Figs. S7, S8). Moreover, a few peptides corresponding to bovine beta-casein (in UV1) and bovidae alpha-S1-casein (in UV2) were identified as well (Supplementary Figs. S4, S13).

Apart from common laboratory and handling contaminants otherwise excluded from this study, we recovered peptides (n = 20 peptide-spectrum matches, PSMs) of *Bos taurus* COL1A1 and COL1A2 in the laboratory blank extraction (Supplementary Table S5). However, the number of cross-contaminated bovine collagen peptides are noticeably fewer than what is detected in both samples (n = 1462 PSMs total). The probable source of this contamination is examined in the discussion.

### Deamidation

Deamidation quantification was also performed on all samples. The extent of deamidation can be used to assess protein degradation and accordingly has often been used to support the ancient origin of the protein fragments recovered as well as the identification of possible contaminants^23,27^. Deamidation is a spontaneous chemical modification that occurs and accumulates over time. It affects asparagine (Asn/N) and glutamine (Gln/Q) amino acids, converting them to aspartic and glutamic acids (Asp/D, Glu/E), respectively^31,32^. However as this rate can be affected by many factors other than time, such as temperature and humidity, as well as by which amino acids surround the N and Q residues^33^, deamidation rates can be used as an indicator of protein damage and aging, but not as a dating tool^32^. Since glutamine deamidates at a much slower rate than asparagine, its deamidation rate seems to be a better indicator of ageing^34^ or sample preservation^35^.

**Figure 3:**
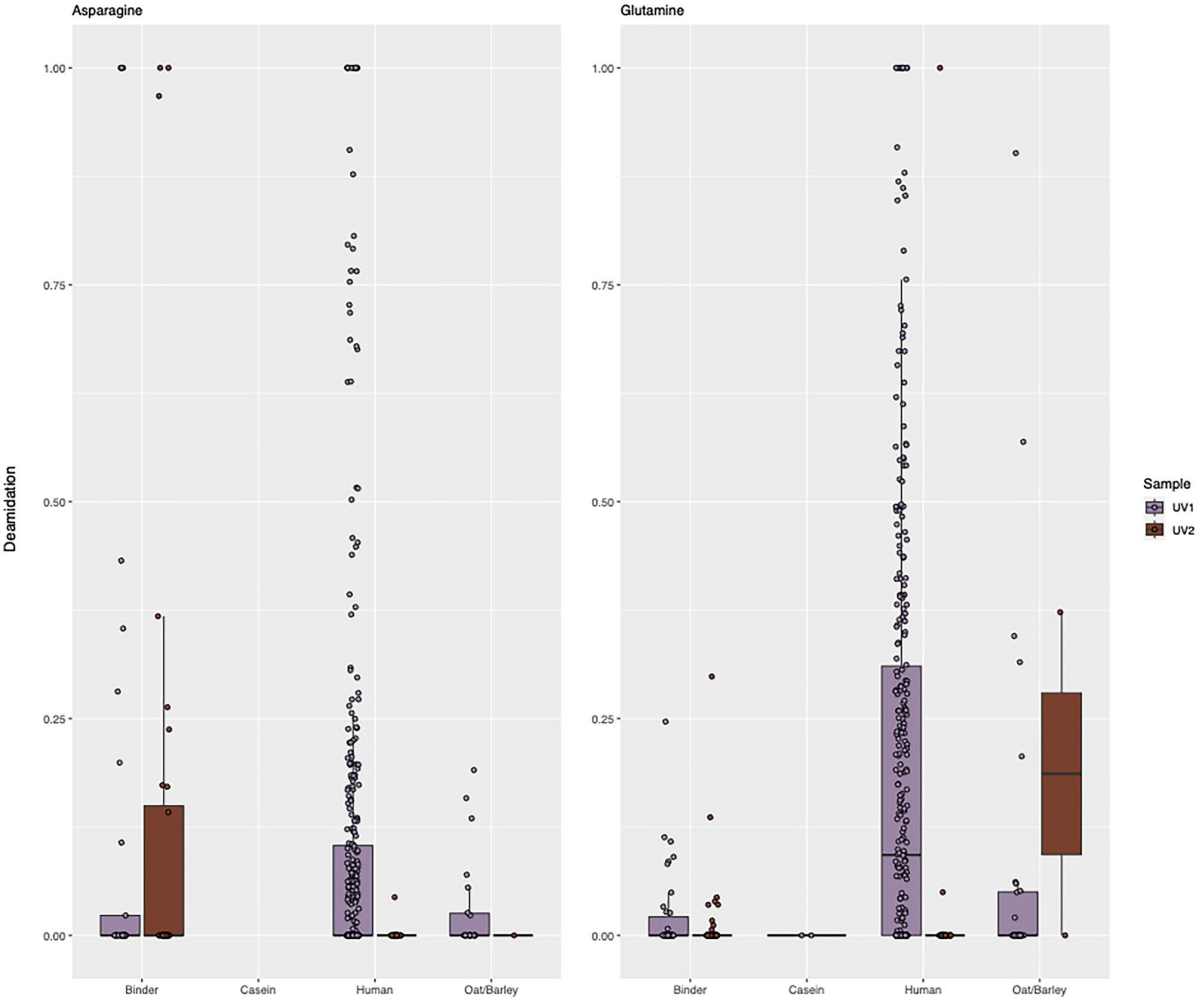
Deamidation of asparagine (left) and glutamine (right) in both samples. Deamidation calculated by deamiDATE 1.0. Each point represents a single amino acid at a certain position within a protein, whereas the boxes represent the distribution of points within each category—binder (which includes all bovidae proteins except caseins), casein, human, and oat/barley. The y-axis represents the remaining amount of deaminating amino acids with 1 representing 100% deamidation.

In our samples, the overall deamidation of the two samples across all proteins is 17% (Q), 11% (N) for UV1, and 3% (Q) and 8% (N) in UV2 (Fig. 3). The total analysis is based on the deamidation status of a total of 955 residues at specific positions. A breakdown of the count of peptides in each condition can be found in Supplementary Table 6. Although the overall deamidation of all proteins in UV2 is according to our expectation of the lower deamidation rate (and thus extent) of glutamine in comparison with asparagine, we see an inconsistency with the deamidation results of UV1 proteins. This is likely due to the large number of saliva proteins in the UV1, which appear unproportionately deamidated, especially for glutamine, based on their assumed modern age (Supplementary Fig. S16). However, it can be seen that deamidation of the salivary proteome already occurs in vivo^36^, sometimes before appearance in the oral cavity (at least for amylase^37^) or after, potentially related to enzymatic activity, including that from oral bacteria^38^. We can confirm this observation when we restrict the deamidation evaluation to just binder proteins (bovine proteins except casein), where the average deamidation for UV1 is 13.3% (N) and 2.3% (Q), and UV2 is 13.5% (N) and 1.4% (Q) (Supplementary Table 7). Furthermore, we speculate that the variation observed in the deamidation patterns of saliva proteins between the two samples could be due to stochasticity introduced by relatively few data points of UV2 compared to UV1 (Supplementary Fig. S16 and Table S6).

The extent of deamidation for the collagen and haemoglobin proteins corresponding to cow in both samples is lower than what we expected in relation to their age. This might have something to do with the way the animal glue was prepared rather than the age of the binder. It is likely that lower denaturing temperatures were used to obtain collagen from skin which might have resulted in lower deamidation rates. Nonetheless, production of animal glue consists of several steps, including (acidic/alkaline) pre-treatment, extraction and dissolution. Differences in these steps could noticeably affect the chemical and mechanical properties of the glue^39^.

In both samples, the site specific deamidation of peptides corresponding to saliva proteins and the oat and barley proteins are comparable, indicating they could potentially be from the same age and/or source (Supplementary Fig. S16). For the caseins, however, the few peptides recovered makes it difficult to be confident of their source and damage state.

## Discussion

Collagen-based animal glues have been widely employed throughout time^40,41^. Raw materials such as skin, bone, and connective tissue from mammals and fish are usually used to prepare animal glues. Through several steps of hydrolysis and denaturation, collagen, an insoluble natural polymer and the main protein component of animal glue, is extracted from such tissues and transforms into soluble gelatin or glue. While bone glues (strong glues) are primarily made from fresh or extracted bones, generally hide glues derive from skins^39^. Depending on their origin and diverse manufacturing processes, animal glues could display different chemical, physical and mechanical properties^42^. Availability and traditional knowledge may also influence the choice of a suitable animal glue made by the artist. Accordingly, the applications of animal glue varies from binding media for pigments^43^, consolidants^44^, and adhesives^45^, to gilding on manuscripts and wooden boards^45^. This diverse use of animal glue, especially in complex structures such as paintings, makes it challenging for researchers to distinguish between the binder and later coatings or consolidation treatments.

In the present study, we discovered cow collagen proteins (COL1A1, COL1A2, COL3A1), implying the use of animal glue as the binder in the distemper paints sampled from Uvdal stave church. The presence of COL3A1 and bovinae keratins indicates that the glue contains cow hide, although it cannot be determined whether this represents pure hide or a mix of hide and bone glue. Bovine keratins could also be incorporated into the paint through the use of a cow hair paint brush, but in context, it is unlikely for COL3A1 to come from a source other than hide. Bovine foetal haemoglobin detected in both samples also suggests that the hide was obtained, at least in part, from calves, due to its absence in cows after a few months of age. This is particularly of interest when the historical record is examined. There is a mention of 12 calf skins for glue production purchased by painter ‘Lars Jensen Mahler’ among other materials, including pigments, in an accounting book from Voss church in 1701^46^. Similarly, in the accounting book ca. 1711 of Jostedal church, 24 calf skins were purchased for the same purpose^12^. It appears that the use of calfskin glue may have been a particular trend in the production of distemper paints for churches at this time in Norway. While we cannot exclude the possibility of a combination of bone and skin being used to produce the animal glue, based on our data and historical records, we believe that the binder is most likely a hide glue made from calfskin.

Regarding the few bovine collagen peptides found in the blank, analysis of their site specific deamidation indicates a more damaged, arguably older, source than those recovered from the samples. Based on the analysis of MS measurement data generated from the samples injected in the instrument before the blank, we believe the contamination originated from an overloaded archaeological cow bone extract and appeared in the extraction blank due to carryover in the nanoLC chromatographic column. This extract was injected into the mass spectrometer just before two nLC column wash injections and the Uvdal stave church extraction blank. We notice a gradual decline in bovine collagen-specific PSMs along these series of MS measurement rounds (Supplementary Table S5). Notably, all collagen peptides identified in the extraction blank are present in at least one of the wash runs, confirming their likely origin from the previous extract. Furthermore, out of 19 collagen 1 peptides in the blank, 13 show higher PSM counts and intensities in the paint samples, indicating that these peptides at least partly derive from the paint samples themselves rather than carryover contamination (the remaining 6 peptides show similar number of PSMs, usually only 1). Nevertheless, we excluded these 19 peptides found in the blank during our assignment. In addition, the other bovine proteins confidently recovered exclusively in the paint samples (bovine collagen 3, keratins, and HBBF) further support our assignment of the binder’s composition.

Our experimental approach did not detect any collagen from sturgeon or other species of fish, nor did we find collagen from any other animal that could have potentially been used in restoration treatments. While accurate determination of the detection limits of our approach would require systematic characterisation and the sacrifice of more cultural heritage material, our results seem to preliminarily confirm that the samples we processed have not been subject to any restoration treatment^19^. The confident detection of proteinaceous material used in documented or undocumented restoration treatments falls beyond the scope of this study and will be investigated in future research.

Unexpectedly, in both samples we detected a large number of human saliva proteins, especially in UV1. While saliva has reportedly been used as a paint binder in certain contexts^47–49^, it is very unlikely that it would be used in late mediaeval Norway, especially considering the amounts required for the large painted area of the church. However, even though the presence of saliva proteins might appear to be peculiar, using one’s own saliva has been one of the most common agents for cleaning a painting’s surface^50^. The so-called tradition of spit cleaning or natural enzymatic cleaning is still in practice^51^ because salivary enzymes such as amylase and lipase can gently help break down surface contamination^52,53^. Alternatively, saliva could have been employed during preliminary examinations by restorers to check the nature of the painting’s binder, or more generally, to test water sensitivity^54^. However, saliva cleaning is not a common practice for the cleaning of wall paintings at NIKU, and would likely not have been attempted due to the fragile nature of the sample. It is, however, also possible that the saliva proteins remain on the surface of the distemper paint boards from an attempt at the time of discovery to remove dust and dirt from the surface of the painting. Saliva is a readily accessible cleaning agent for the person in the field, and it would be a natural impulse. Therefore, we consider this the most likely source of the saliva contamination.

It should still be emphasised that the presence of saliva creates difficulties for proteomic analysis. The presence of highly abundant modern proteins can mask the recovery of lower abundance, endogenous proteins, due to the nature of the data-dependent mass spectrometry methods employed here, i.e. selecting the topmost abundant peaks for sequencing. Although this can possibly hinder the recovery of binder proteins, their identification in this case was not hampered. This would likely make more of an impact on older, more degraded samples. However, the introduction of saliva also impedes the identification of the binder’s composition, since it may contain proteins consumed by the individual which are also plausible ingredients in the compounds used in producing an artwork. In this context, the presence of oat and barley proteins in UV1 are likely to be a result of their consumption by the individual who produced the saliva. However, in other contexts where starch glues or additives may be encountered, the presence of saliva would cause doubt in the provenance of these proteins. Such is the case for the casein proteins discovered in both samples. Since these proteins derive from milk, it is impossible to know if they are present due to the saliva contamination or if they were used in the production of the artwork. In artworks, caseins can be used alone as binders and glues, but are also sometimes additives to these products.

Casein use for paint binder preparation is not recorded in historical instructions from the 17th and 18th century in Norway. Kristian Kildal (1906–1988) describes the use of casein by folk art painters in certain areas of Norway in the eighteenth century but does not give any reference for this information^55^. In addition, recent studies on distemper paints do not confirm the presence of casein in stave churches^19,56^. If the caseins identified in our samples are genuinely from the painted boards, we believe that they would be an additive to the first layer (underpaint). Casein was sometimes added to the binder of the white underpaint in an attempt to decrease the water solubility^57^, making it easier to apply the subsequent paint layers. However, since these proteins may also come from contamination, we cannot definitively make any claim as to the cause of their presence. Additional samples from Udval without saliva contamination would be needed to investigate this further. However, the current study is focused on the identification of the original binder of the distemper paint for future conservation considerations, so no further samples were taken. On the other hand, our study demonstrates the importance of considering possible sources of post-application contamination when examining the composition of historical paint samples. We recommend that those handling these objects are made aware of the possible implications of using saliva in hindering future palaeoproteomic analysis.

## Conclusion

Palaeoproteomics has proven to be a powerful tool in detecting and characterising proteins in archaeological contexts, even when these materials are degraded and complex. In the present study, in spite of the presence of a large number of contaminants, we were able to confidently identify several tissues and contributing species present within the binder. However, it must be noted that the unexpected presence of saliva in the samples was problematic in interpreting the data. Therefore, it is recommended to be mindful when employing techniques that could introduce potential contaminants to the object.

This is the first time, to our knowledge, that paint samples from stave churches in Norway were examined for characterisation of their binding media in such detail. Further research should focus on studying samples with palaeoproteomic analysis, from a variety of Norwegian stave churches to investigate whether or not there was an established practice for binder production. This could further improve our knowledge about the artisans’ choice of materials during this period and standard recipes.

### Supplementary Information

Supplementary Information.

## Data Availability

The mass spectrometry proteomics data have been deposited to the ProteomeXchange Consortium (http://proteomecentral.proteomexchange.org) via the PRIDE^58^ partner repository with the dataset identifier PXD043606.
