# Supplementary material for: Palaeoproteomic identification of the original binder and modern contaminants in distemper paints from Uvdal stave church, Norway
Source: Sci Rep. 2024 Jun 4;14:12858. doi: 10.1038/s41598-024-63455-4 (PMC11150381; doi:10.1038/s41598-024-63455-4)
Supplement: Supplementary file 1 — Supplementary Information. [file 41598_2024_63455_MOESM1_ESM.pdf]

SUPPLEMENTARY INFORMATION

Palaeoproteomic identification of the original binder and modern contaminants in distemper paints from Uvdal stave church, Norway

Zahra Haghighi[1+]*, Meaghan Mackie[2,3,4+]*, Anne Apalnes Ørnhøi[5], Abigail Ramsøe[2], Tone Marie Olstad[5], Simon James Armitage[1,6], Christopher Stuart Henshilwood[1,7], Enrico Cappellini[2]*

[1]SFF Centre for Early Sapiens Behaviour (SapienCE), University of Bergen, Bergen, Norway

[2]The Globe Institute, University of Copenhagen, Copenhagen, Denmark

[3]School of Archaeology, University College Dublin, Dublin, Ireland

[4]ArchaeoBiomics, Department of Life Sciences and Systems Biology, University of Turin, Turin, Italy

[5]The Norwegian Institute for Cultural Heritage Research (NIKU), Oslo, Norway

[6]Department of Geography, Royal Holloway University of London, Egham, Surrey, UK

[7]Evolutionary Studies Institute, University of the Witwatersrand, Johannesburg, South Africa

*Corresponding authors respectively: zahra.haghighi@uib.no, meaghan@palaeome.org, ecappellini@sund.ku.dk

+These authors contributed equally

FIGURES

Selected MS/MS Peptide Spectra relevant for Species and Protein Identification

The spectra were made using Interactive Peptide Spectral Annotator[7].

Key

Lowercase letters in the amino acid sequence are indicative of post-translational modifications:

c- carbamidomethylation

m-methionine oxidation

n-asparagine deamidation

p-hydroxyproline

q-glutamine deamidation; pyro-glu

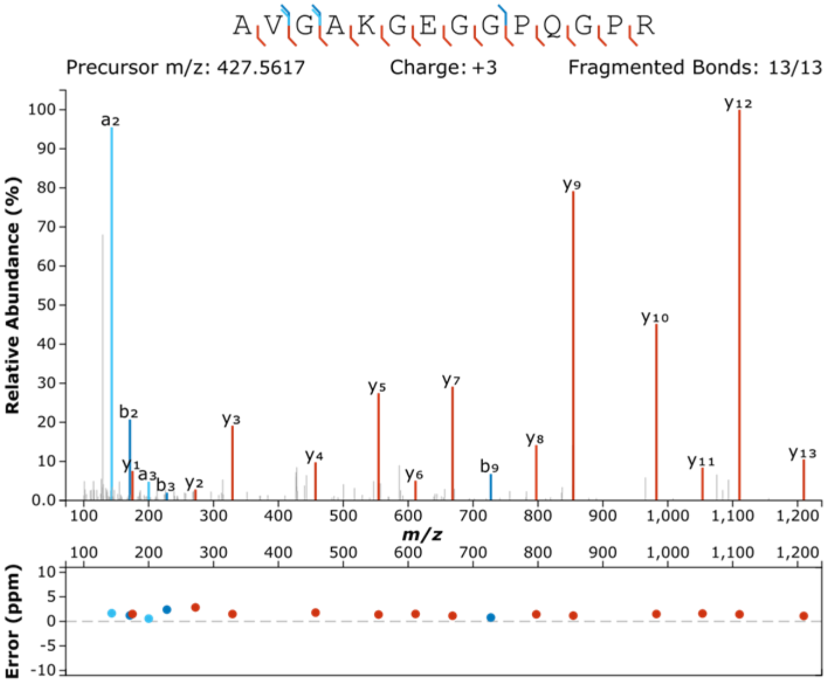

Supplementary Fig. S1- Sample UV1, Bovinae Collagen 1 alpha 1, scan 3042

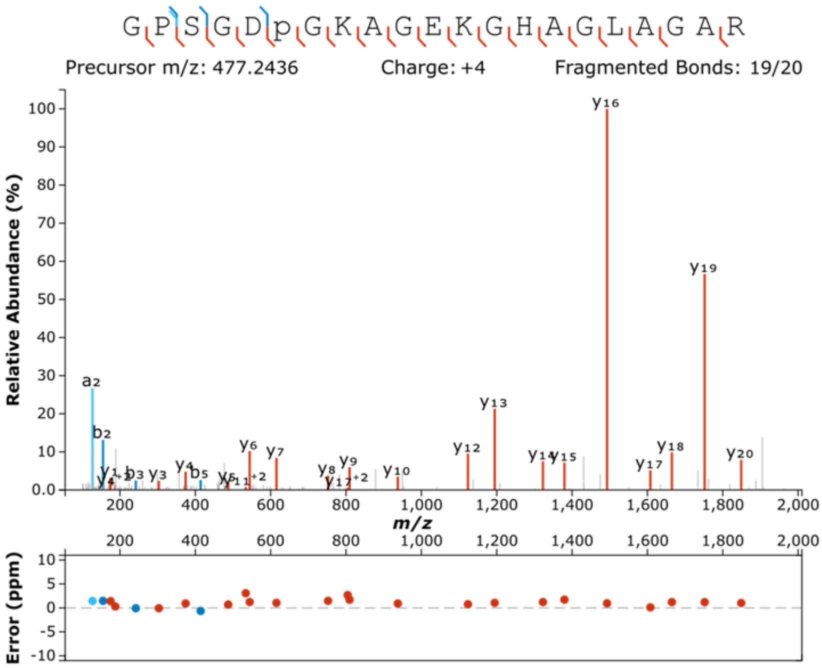

Supplementary Fig. S2- Sample UV1, Bovinae Collagen 1 alpha 2, scan 3237

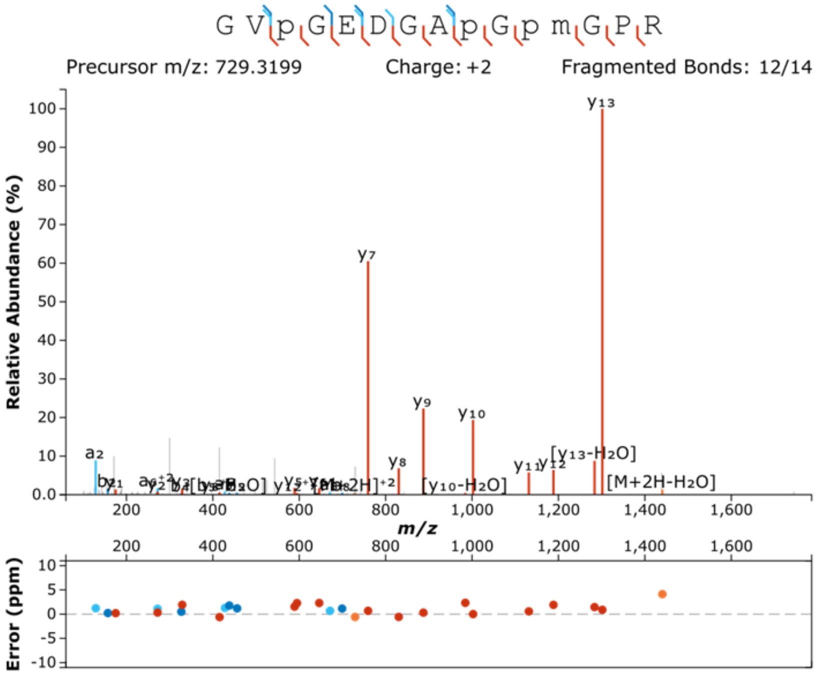

Supplementary Fig. S3- Sample UV1, *Bos* sp. and *Bison bison bison* Collagen 3, scan 5993

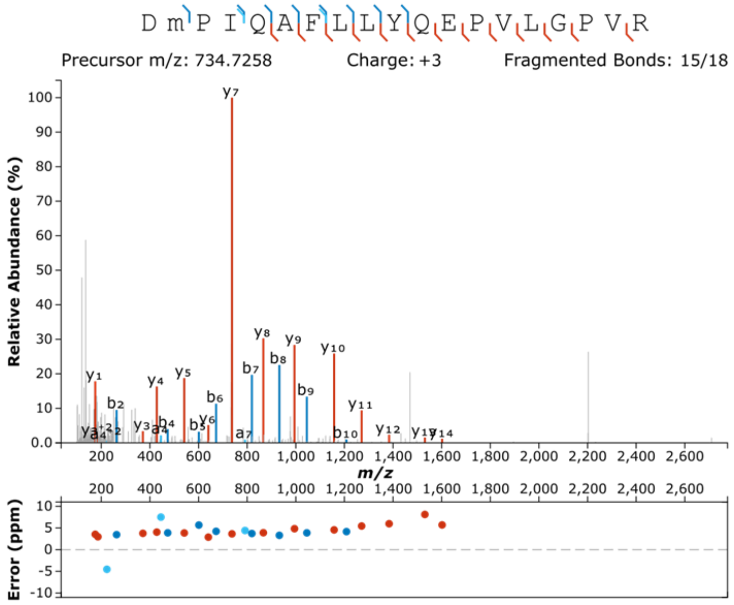

Supplementary Fig. S4- Sample UV1, Bovidae Beta-casein, scan 23193

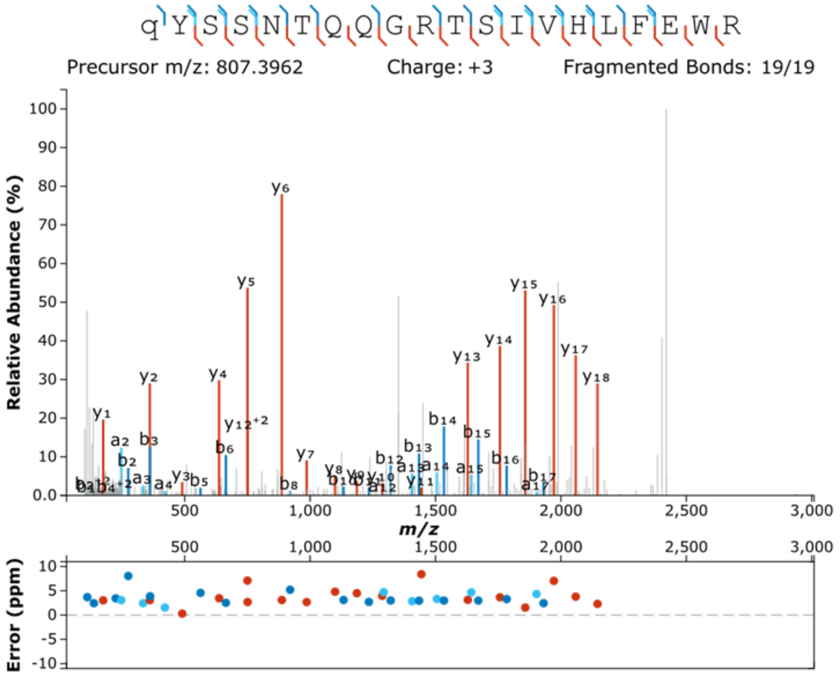

Supplementary Fig. S5- Sample UV1, *Homo sapiens* Alpha-amylase 1A, scan 19112

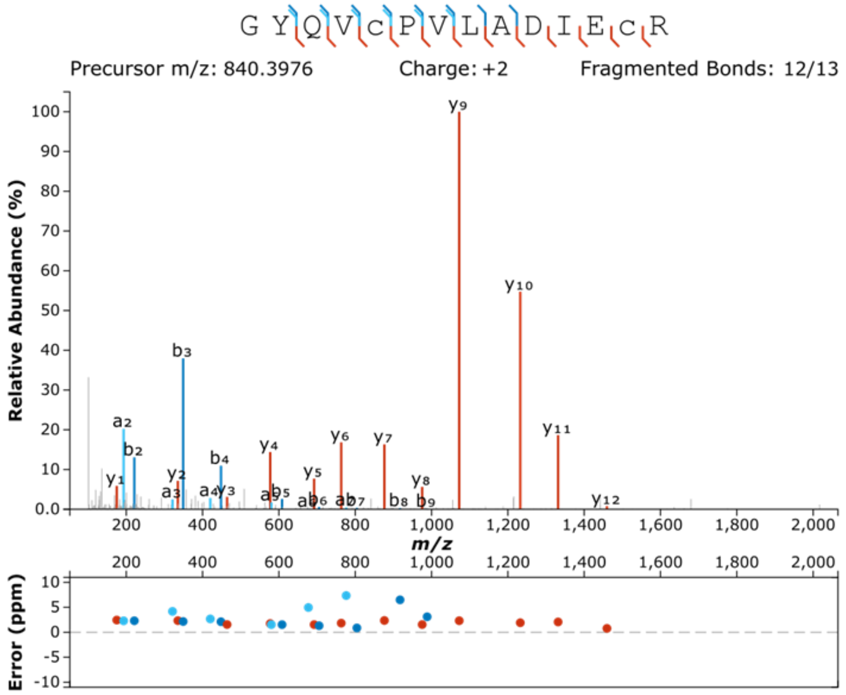

Supplementary Fig. S6- Sample UV1, *Homo sapiens* Mucin 5B, scan 17386

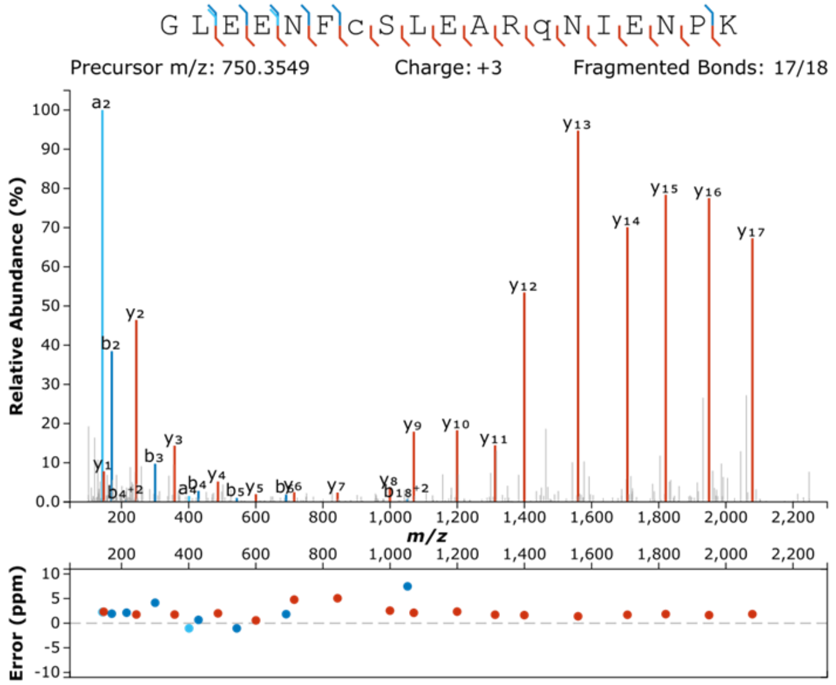

Supplementary Fig. S7- Sample UV1, *Avena sativa* 12S seed storage globulin 1, scan 14566

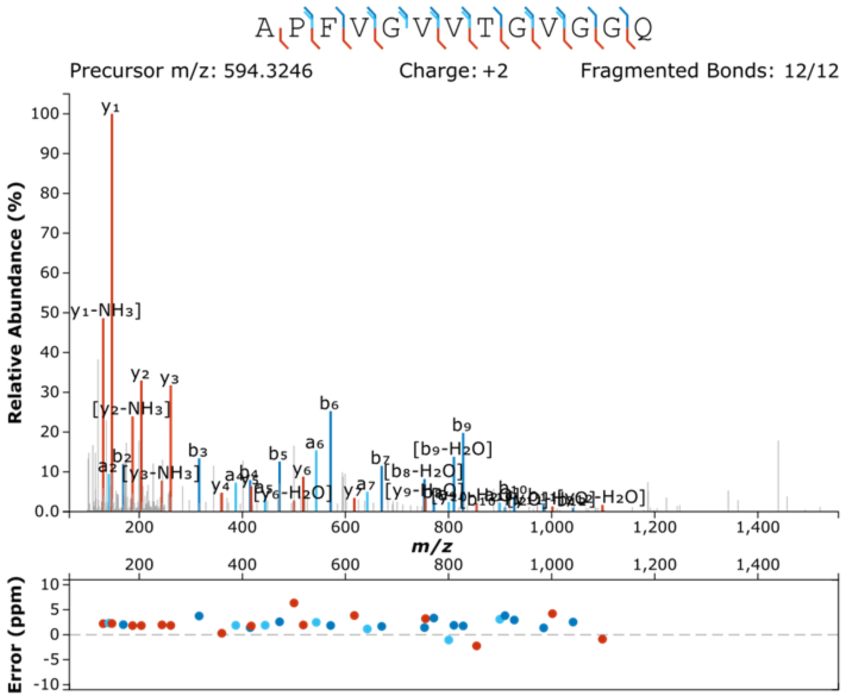

Supplementary Fig. S8- Sample UV1, Gamma-hordein-1 *Hordeum vulgare, Triticum aestivum*, scan 17022

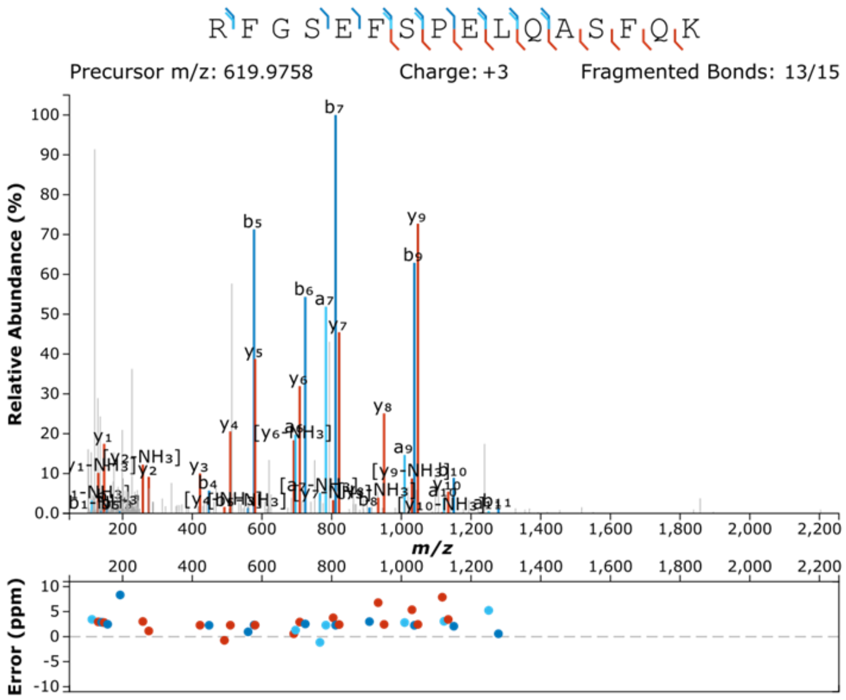

Supplementary Fig. S9- Sample UV1, Bovinae Hemoglobin fetal subunit beta, scan 14720

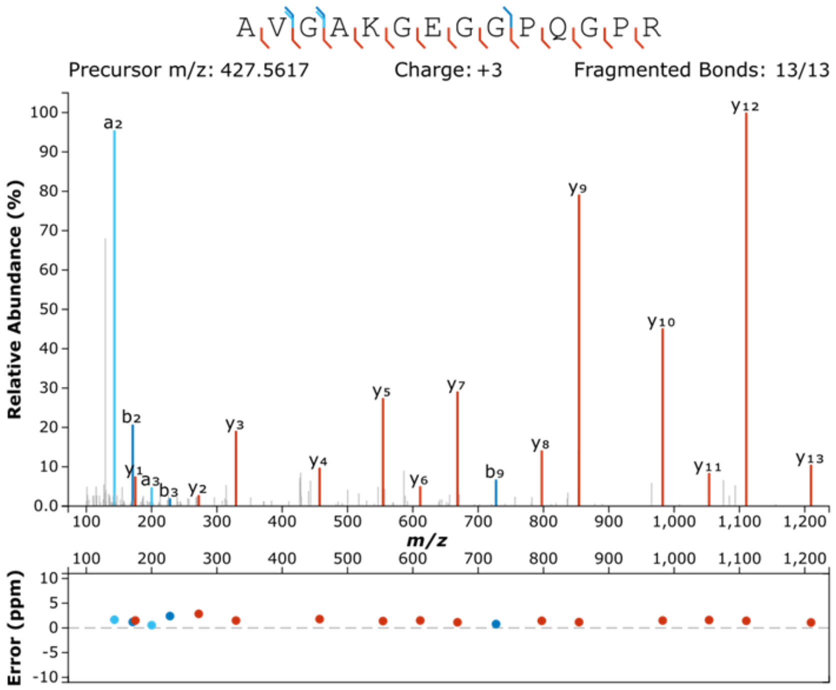

Supplementary Fig. S10- Sample UV2, Bovinae Collagen 1 alpha 1, scan 2800

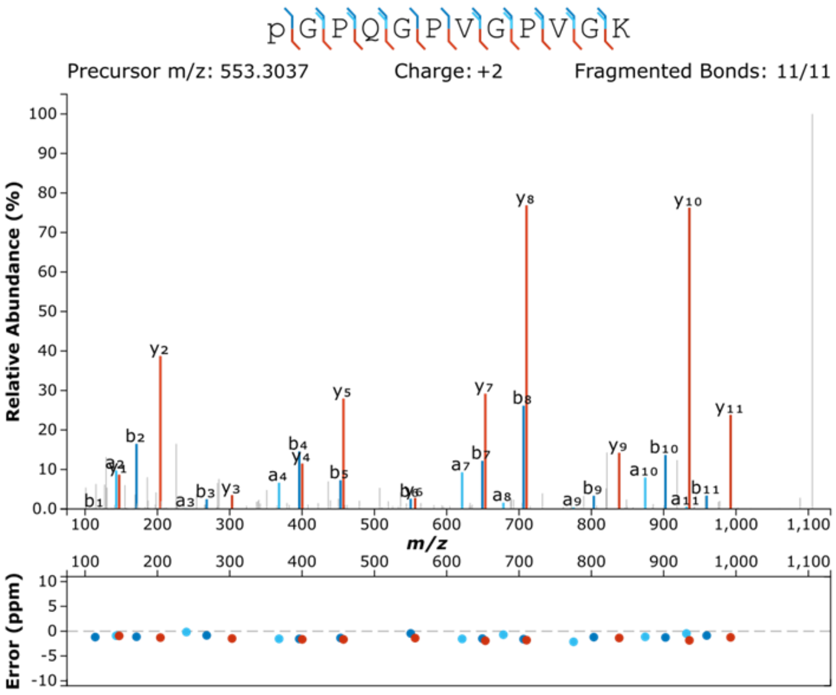

Supplementary Fig. S11- Sample UV2, Bovinae Collagen 1 alpha 2, scan 11195

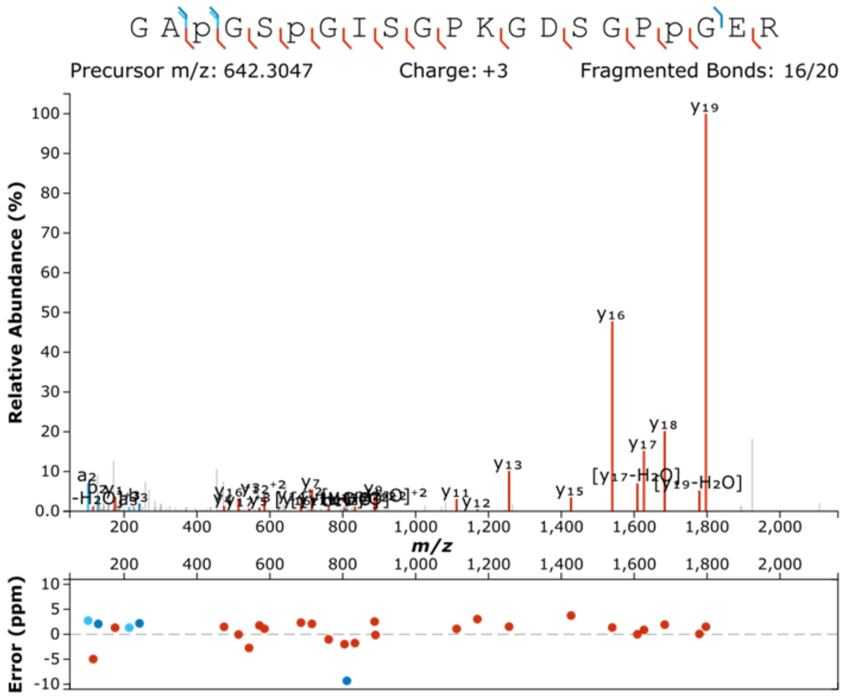

Supplementary Fig. S12- Sample UV2, *Bos* sp. and *Bison bison bison* Collagen 3, scan 4789

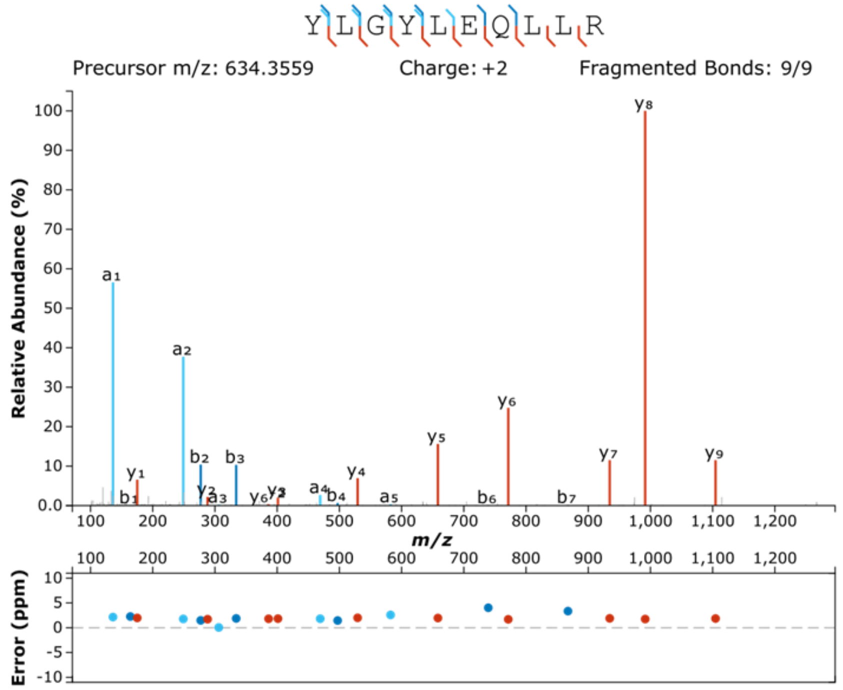

Supplementary Fig. S13- Sample UV2, Bovinae Alpha-S1-casein, scan 21974

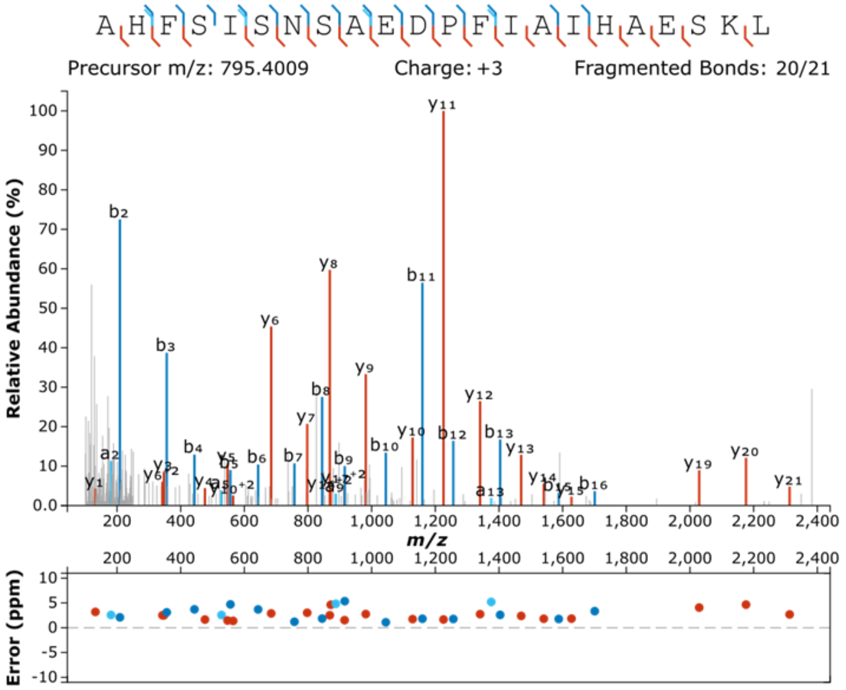

Supplementary Fig. S14- Sample UV2, *Homo sapiens* Alpha-amylase 1A, scan 18299

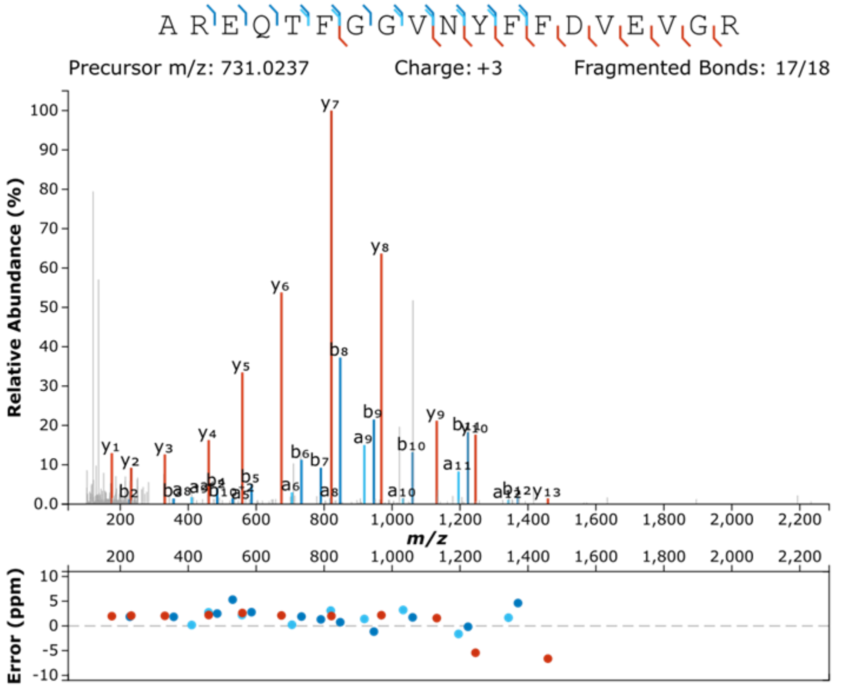

Supplementary Fig. S15- Sample UV2, *Homo sapiens* Cystatin-S, scan 20735

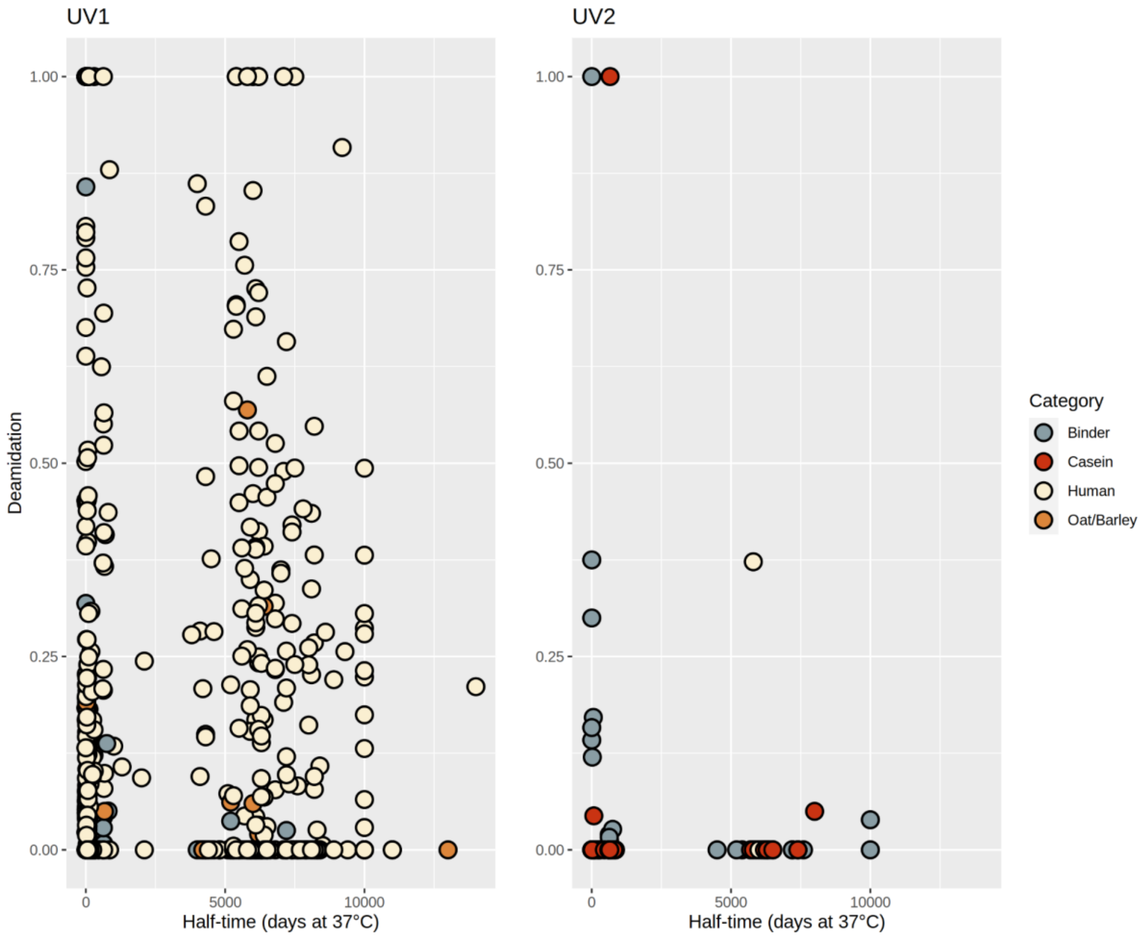

Supplementary Fig. S16- Site-specific deamidation of the two samples, UV1 (left) and UV2 (right). Each point represents a specific three residue combination containing a deamidation site in the middle. Half-time is shown on the x-axis, and the relative proportion of non-deamidated residues is shown on the y-axis, with 1 representing 100% deamidation.

TABLES

Table S1- UV1 protein summary.

**Protein no**	**Leading Razor Protein**	**Species Specificity of Peptides Recovered**	**No. of Identified Razor+ Unique Peptides (semi-tryptic)**	**% Coverage (not razor)**	**No. of MS2 spectra (razor)**
**1**	P02453 Collagen alpha-1(I) chain	Bovinae	281	55.4	391
**2**	P0DUB6 Alpha-amylase 1A	*Homo sapiens*	221	94.1	552
**3**	P02465 Collagen alpha-2(I) chain	Bovinae	203	44.2	259
**4**	Q9HC84 Mucin-5B	*Homo sapiens*	156	21	294
**5**	P02788 Lactotransferrin	*Homo sapiens*	113	73.2	254
**6**	P01876 Immunoglobulin heavy constant alpha 1	*Homo sapiens*	110	84.4	204
**7**	Q8TDL5 BPI fold-containing family B member 1	*Homo sapiens*	92	70	167
**8**	P02768 Albumin	*Homo sapiens, Pongo abelii*	92	82.6	182
**9**	P04258 Collagen alpha-1(III) chain	*Bos* sp., *Bison bison bison*	74	32.2	112
**10**	Q25010 Actin, cytoplasmic A3a	aspecific	73	74.5	116

Table S1_*Continued.*

**Protein no**	**Leading Razor Protein**	**Species Specificity of Peptides Recovered**	**No. of Identified Razor+ Unique Peptides (semi-tryptic)**	**% Coverage (not razor)**	**No. of MS2 spectra (razor)**
**11**	P01833 Polymeric immunoglobulin receptor	*Homo sapiens*	67	43.6	137
**12**	P61626 Lysozyme C	Homininae	61	82.4	175
**13**	P05164 Myeloperoxidase	Homininae, *Canis lupus familiaris*	59	47.2	131
**14**	Q9UGM3 Deleted in malignant brain tumors 1 protein	*Homo sapiens*	56	41.9	114
**15**	P01036 Cystatin-S	*Homo sapiens*	48	71.6	142
**16**	Q8N4F0 BPI fold-containing family B member 2	*Homo sapiens*	40	46.3	57
**17**	P06702 Protein S100-A9	Homininae	39	71.1	100
**18**	P12615 12S seed storage globulin 1	*Avena sativa*	36	41.3	87
**19**	P01834 Immunoglobulin kappa constant	aspecific	35	77.6	64
**20**	P31025 Lipocalin-1	*Homo sapiens*	31	76.1	62
**21**	P01871 Immunoglobulin heavy constant mu	*Homo sapiens*	30	33.3	50
**22**	Q5REL2 Annexin A1	Boreoeutheria	29	56.9	52

Table S1_*Continued.*

**Protein no**	**Leading Razor Protein**	**Species Specificity of Peptides Recovered**	**No. of Identified Razor+ Unique Peptides (semi-tryptic)**	**% Coverage (not razor)**	**No. of MS2 spectra (razor)**
**23**	P08246 Neutrophil elastase	*Homo sapiens*	26	51.3	51
**24**	P08311 Cathepsin G	*Homo sapiens*	26	59.2	77
**25**	P01591 Immunoglobulin J chain	Hominoidea	24	66.7	37
**26**	P11684 Uteroglobin	*Homo sapiens, Pan* sp.	24	63.7	58
**27**	P84049 Histone H4	aspecific	24	60.2	50
**28**	P05109 Protein S100-A8	Homininae	20	51.6	35
**29**	Q9Y6R7 IgGFc-binding protein	Homininae	20	6.9	28
**30**	P25311 Zinc-alpha-2-glycoprotein	Homininae	18	41.9	29
**31**	P10854 Histone H2B type 1-M	aspecific	18	61.9	47
**32**	P52480 Pyruvate kinase PKM	aspecific	17	31.1	27
**33**	Q6FI13 Histone H2A type 2-A	aspecific	16	65.4	53
**34**	P20160 Azurocidin	*Homo sapiens*	15	38.2	26
**35**	Q5R6Y1 Alpha-enolase	*Homo sapiens*	15	32.5	23
**36**	Q9U281 Histone H3.3 type 2	aspecific	15	55.1	32

Table S1_*Continued.*

**Protein no**	**Leading Razor Protein**	**Species Specificity of Peptides Recovered**	**No. of Identified Razor+ Unique Peptides (semi-tryptic)**	**% Coverage (not razor)**	**No. of MS2 spectra (razor)**
**37**	P01024 Complement C3	*Homo sapiens*	14	11.7	18
**38**	P09228 Cystatin-SA	*Homo sapiens*	14	74.5	25
**39**	Q9NP55 BPI fold-containing family A member 1	*Homo sapiens*	14	51.6	26
**40**	P29401 Transketolase	Catarrhini	13	23.1	18
**41**	P84198 Vimentin	Amniota	13	30.7	26
**42**	P23280 Carbonic anhydrase 6	*Homo sapiens*	12	36.4	25
**43**	P80188 Neutrophil gelatinase-associated lipocalin	*Homo sapiens*	12	63.6	22
**44**	P01780 Immunoglobulin heavy variable 3-7	aspecific	11	42.7	18
**45**	P04350 Tubulin beta-4A chain	aspecific	11	21.6	16
**46**	P01861 Immunoglobulin heavy constant gamma 4	aspecific	10	28.7	16
**47**	P10719 ATP synthase subunit beta, mitochondrial	aspecific	9	28.4	12
**48**	P30740 Leukocyte elastase inhibito	*Homo sapiens*	9	32.2	20
**49**	Q96DA0 Zymogen granule protein 16 homolog B	*Homo sapiens*	9	34.6	19

Table S1_*Continued.*

**Protein no**	**Leading Razor Protein**	**Species Specificity of Peptides Recovered**	**No. of Identified Razor+ Unique Peptides (semi-tryptic)**	**% Coverage (not razor)**	**No. of MS2 spectra (razor)**
**50**	P01023 Alpha-2-macroglobulin	Catarrhini	8	6.6	12
**51**	O57672 Glyceraldehyde-3-phosphate dehydrogenase	aspecific	8	24.8	11
**52**	P68361 Tubulin alpha-1B chain	aspecific	8	21.7	12
**53**	Q3SX09 Hemoglobin fetal subunit beta	Bovinae	7	39.3	11
**54**	P02769 Albumin *	Pecora	7	19.6	7
**55**	Q9Z1P2 Alpha-actinin-1	aspecific	7	8.7	11
**56**	P02662 Alpha-S1-casein *	*Bos taurus, Bubalus bubalis* **	6	20.6	7
**57**	P01966 Hemoglobin subunit alpha	Bovinae	6	43	10
**58**	P02787 Serotransferrin	Simiiformes	6	12.9	8
**59**	Q76LA0 Cystatin-B	Catarrhini	6	48	11
**60**	P17990 Gamma-hordein-1	*Hordeum vulgare, Triticum aestivum*	4	19.3	4
**61**	Q92176 Coronin-1A	Eutheria	4	10.8	5
**62**	ENSBTAP00000038253 Keratin type II cytoskeletal 1	*Bos* sp., *Bison bison bison*	3	21.3	4

Table S1_*Continued.*

Protein no	Leading Razor Protein	Species Specificity of Peptides Recovered	No. of Identified Razor+ Unique Peptides (semi-tryptic)	% Coverage (not razor)	No. of MS2 spectra (razor)
**63**	Q3ZBD7 Glucose-6-phosphate isomerase	aspecific	3	4.5	5
**64**	P01700 Immunoglobulin lambda variable 1-47	*Homo sapiens, Mus musculus*	3	29.1	5
**65**	P04075 Fructose-bisphosphate aldolase A	aspecific	3	15.4	6
**66**	P19181 Ig heavy chain V region 5A	aspecific	3	43.1	9
**67**	P33048 Beta-casein *	Bovidae **	3	11.7	4
**68**	P02070 Hemoglobin subunit beta	Artiodactyla	2	31	3
**69**	Q3SZR3 Alpha-1-acid glycoprotein	*Bos* sp., *Rupicapra rupicapra*	2	13.4	2
**70**	P0CN30 Elongation factor 1-alpha	aspecific	2	5.9	7
**71**	Q5R5A3 Antithrombin-III	Primates	2	4.7	3
**72**	Q6P698 Plastin-2	aspecific	2	3.5	2

* - It should be noted that this is a possible lab contamination, however this protein was not found in the blank. While it should be observed with caution, this is assumed to be authentic, also supported by the presence of other bovine proteins.
** - Several bovine milk proteins have exact replicas in the NCBI database with Staphylococcaceae, especially Jeotgalicoccus sp.. These are observed with caution, but it is unlikely that these are correct (likelihood of at least 3 exactly the same proteins is low), and are more likely some form of genome contamination for these species.

Table S2- UV1 specific peptides.

**Protein ID**	***Leading Razor Protein***	**Peptide**	**Species**	**Start position**	**End position**	**Score**	**No. of MS2**
**1**	*P02453 Collagen alpha-1(I) chain*	PGAVGAKGEGGPQGPR	Bovinae	345	360	96.489	2
		AVGAKGEGGPQGPR	Bovinae	347	360	125.53	1
		VGAKGEGGPQGPR	Bovinae	348	360	62.732	1
		GEGGPQGPRGSEGPQGVR	Bovinae	352	369	110.37	1
**2**	*P0DUB6 Alpha-amylase 1A*	QYSSNTQQGRTSIVHLFEWR	*Homo sapiens*	16	35	215.88	1
**3**	*P02465 Collagen alpha-2(I) chain*	GPSGDPGKAGEKGHAGLAGAR	Bovinae	497	517	127.17	1
		SGETGASGPPGFVGEKG	Bovinae	829	845	153.81	1
		AGPVGAAGAPGPQGPVGPVGK	Bovinae	952	972	84.829	1
		AAGAPGPQGPVGPVGK	Bovinae	957	972	146.11	1
		GAPGPQGPVGPVGK	Bovinae	959	972	111.65	1

Table S2-*Continued.*

**Protein ID**	***Leading Razor Protein***	**Peptide**	**Species**	**Start position**	**End position**	**Score**	**No. of MS2**
**3**	*P02465 Collagen alpha-2(I) chain*	APGPQGPVGPVGK	Bovinae	960	972	75.387	1
		GPAGPSGPAGKDGRIGQPG	Bovinae	1052	1070	110.37	1
		GRIGQPGAVGPAGIR	Bovinae	1064	1078	67.136	1
**4**	*Q9HC84 Mucin-5B*	QRGYQVCPVLADIECR	*Homo sapiens*	1366	1381	231.06	4
		GYQVCPVLADIECR	*Homo sapiens*	1368	1381	214.26	5
		QVCPVLADIECR	*Homo sapiens*	1370	1381	89.548	1
		AAQLPDMPLEELGQQVDCDRMR	*Homo sapiens*	1382	1403	115.66	3
		NHYCTASATAAAAR	*Homo sapiens*	5118	5131	145.52	1
		CTASATAAAAR	*Homo sapiens*	5121	5131	91.867	1
		NGVLVSVLGTTTMR	*Homo sapiens*	5174	5187	95.538	4

Table S2-*Continued.*

Protein ID	*Leading Razor Protein*	Peptide	Species	Start position	End position	Score	No. of MS2
**5**	*P02788 Lactotransferrin*	FGRNGSDCPDKFCLFQSETK	*Homo sapiens*	639	658	212.39	3
		NGSDCPDKFCLFQSETK	*Homo sapiens*	642	658	108.47	1
		YLGPQYVAGITNLK	*Homo sapiens*	681	694	176.62	2
		YLGPQYVAGITNLKK	*Homo sapiens*	681	695	160.86	4
**6**	*P01876 Immunoglobulin heavy constant alpha 1*	DLYTTSSQLTLPATQCLAGK	*Homo sapiens, Pan troglodytes*	62	81	227.47	2
		SVSSVLPGCAEPWNHGK	*Homo sapiens, Gorilla gorilla gorilla*	184	200	94.544	1
**7**	*Q8TDL5 BPI fold-containing family B member 1*	TIVEFHMTTEAQATIR	*Homo sapiens*	127	142	132.59	4
		EFHMTTEAQATIR	*Homo sapiens*	130	142	215.53	3
		MTTEAQATIR	*Homo sapiens*	133	142	126.07	1

Table S2-*Continued.*

**Protein ID**	**Leading Razor Protein**	**Peptide**	***Species***	**Start position**	**End position**	**Score**	**No. of MS2**
7	*Q8TDL5 BPI fold-containing family B member 1*	TTEAQATIR	*Homo sapiens*	134	142	66.692	1
		AAVAAVLSPEEF	*Homo sapiens*	291	302	97.093	1
		AAVAAVLSPEEFMV	*Homo sapiens*	291	304	141.52	1
		AAVAAVLSPEEFMVLL	*Homo sapiens*	291	306	118.18	1
		AAVAAVLSPEEFMVLLDSVLPESA	*Homo sapiens*	291	314	182.1	1
		VAAVLSPEEFMVLLDSVLPESAHR	*Homo sapiens*	293	316	220.25	1
		AAVLSPEEFMVLLDSVLPESAHR	*Homo sapiens*	294	316	188.8	4
		AVLSPEEFMVLLDSVLPESAHR	*Homo sapiens*	295	316	171.59	3
		VLSPEEFMVLLDSVLPESAHR	*Homo sapiens*	296	316	168.79	4
		LSPEEFMVLLDSVLPESAHR	*Homo sapiens*	297	316	202.39	1

Table S2-*Continued.*

Protein ID	Leading Razor Protein	Peptide	*Species*	Start position	End position	Score	No. of MS2
**7**	*Q8TDL5 BPI fold-containing family B member 1*	PEEFMVLLDSVLPESAHR	*Homo sapiens*	299	316	108.49	1
		EEFMVLLDSVLPESAHR	*Homo sapiens*	300	316	145	1
		MVLLDSVLPESAHR	*Homo sapiens*	303	316	64.675	1
		LLDSVLPESAHR	*Homo sapiens*	305	316	146.48	2
		SVLPESAHR	*Homo sapiens*	308	316	116.25	1
**8**	*P02768ALBU Albumin*	ALVLIAFAQYLQQCPFEDHVK	*Homo sapiens, Pongo abelii*	45	65	155.28	1
**9**	*P04258 Collagen alpha-1(III) chain*	GVPGE(N/D)GAPGPMGPR	*Bos* sp., *Bison bison bison*	132	146	113.63	2
		GAPGSPGISGPKGDSGPPGER	*Bos* sp., *Bison bison bison*	912	932	105.67	1
**10**	*Q25010 Actin, cytoplasmic A3a*	*aspecific*					

Table S2-*Continued.*

**Protein ID**	***Leading Razor Protein***	**Peptide**	***Species***	**Start position**	**End position**	**Score**	**No. of MS2**
**11**	*P01833 Polymeric immunoglobulin receptor*	LDIQGTGQLLFSVVINQLR	*Homo sapiens*	193	211	191.19	1
		QLLFSVVINQLR	*Homo sapiens*	200	211	116.52	1
		YEDLRGSVTFHCALGPEVANVAK	*Homo sapiens*	246	268	159.16	2
		SPTVVKGVAGGSVAVLCPYNR	*Homo sapiens*	355	375	150.91	1
		SPTVVKGVAGGSVAVLCPYNRK	*Homo sapiens*	355	376	232.29	2
		AFVNCDENSRLVSLTLNLVTR	*Homo sapiens*	516	536	221.82	3
		LVSLTLNLVTR	*Homo sapiens*	526	536	213.03	19
		VLDSGFREIENK	*Homo sapiens*	586	597	88.803	1
**12**	*P61626 Lysozyme C*	ATNYNAGDRSTDYGIFQINSR	Eutheria (only Hominidae are Homininae)	60	80	507.88	10

Table S2-*Continued.*

Protein ID	Leading Razor Protein	Peptide	Species	Start position	End position	Score	No. of MS2
**12**	*P61626 Lysozyme C*	HLSCSALLQDNIADAVACAK	Hominidae, *Panthera pardus*	96	115	430.31	6
		SCSALLQDNIADAVACAK	Hominidae, *Panthera pardus*	98	115	156.24	2
		SALLQDNIADAVACAK	Hominidae, *Panthera pardus*	100	115	252.42	11
		SALLQDNIADAVACAKR	Hominidae, *Panthera pardus*	100	116	119.99	2
		NRCQNRDVRQYVQGCGV	Hominidae, *Panthera pardus*	132	148	210.91	9
**13**	*P05164 Myeloperoxidase*	FCGLPQPETVGQLGTVLR	Homininae, *Canis lupus familiaris*	605	622	185.99	7

Table S2-*Continued.*

**Protein ID**	***Leading Razor Protein***	**Peptide**	***Species***	**Start position**	**End position**	**Score**	**No. of MS2**
**14**	*Q9UGM3 Deleted in malignant brain tumors 1 protein*	QLGCGWAMLAPGNAR	*Homo sapiens, Macaca fascicularis*	534	548	70.412	1
		INLGFSNLK	Homininae	1804	1812	103.42	1
		SGCVRDDTYGPYSSPSLR	Homininae	2321	2338	163.45	1
**15**	*P01036 Cystatin-S*	AREQTFGGVNYFFDVEVGR	*Homo sapiens*	73	91	178.52	13
**16**	*Q8N4F0 BPI fold-containing family B member 2*	ALQVTVPHFLDWSGEALQPTR	*Homo sapiens, Gorilla gorilla gorilla*	48	68	158.33	2
		GLSQQLFDSALLLLQK	*Homo sapiens, Pan* sp.	258	273	299.2	2
**17**	*P06702 Protein S100-A9*	DLQNFLKKENKNEK	Homininae	44	57	87.447	1

Table S2-*Continued.*

**Protein ID**	***Leading Razor Protein***	**Peptide**	***Species***	**Start position**	**End position**	**Score**	**No. of MS2**
**18**	*P12615 12S seed storage globulin 1*	FDRLQAFEPLRQVR	*Avena sativa*	47	60	197.14	7
		LQAFEPLRQVR	*Avena sativa*	50	60	197.06	6
		RVIEPQGLLLPQYHNAPGLVY	*Avena sativa*	86	106	114.07	1
		VIEPQGLLLPQYHNAPGLVYILQGR	*Avena sativa*	87	111	221.82	4
		GLEENFCSLEARQNIENPK	*Avena sativa*	318	336	352.41	3
		NFPTLNLVQMSATR	*Avena sativa*	355	368	137.16	2
		ARVQVVNNHGQTVFNDILR	*Avena sativa*	396	414	294.65	3
		ARVQVVNNHGQTVFNDILRR	*Avena sativa*	396	415	310.23	2
		VQVVNNHGQTVFNDILR	*Avena sativa*	398	414	335.07	5
		VQVVNNHGQTVFNDILRR	*Avena sativa*	398	415	284.72	3

Table S2-*Continued.*

**Protein ID**	**Leading Razor Protein**	**Peptide**	***Species***	**Start position**	**End position**	**Score**	**No. of MS2**
**18**	*P12615 12S seed storage globulin 1*	RGQLLIIPQHYVVLK	*Avena sativa*	415	429	161.76	3
		KAEREGCQYISFK	*Avena sativa*	430	442	180.54	1
		NLKNNRGEEFGAFTPK	*Avena sativa*	480	495	160.64	6
		NNRGEEFGAFTPK	*Avena sativa*	483	495	168.98	2
**19**	*P01834 Immunoglobulin kappa constant*	*aspecific*					
**20**	*P31025 Lipocalin-1*	KLVGRDPKNNLEALEDFEK	*Homo sapiens*	132	150	196.76	2
**21**	*P01871 Immunoglobulin heavy constant mu*	VSVFVPPRDGFFGNPRK	*Homo sapiens, Aotus nancymae*	113	129	122.75	3
		GVALHRPDVYLLPPAR	*Homo sapiens, Pongo abelii*	323	338	155.92	2

Table S2-*Continued.*

**Protein ID**	**Leading Razor Protein**	**Peptide**	***Species***	**Start position**	**End position**	**Score**	**No. of MS2**
**21**	*P01871 Immunoglobulin heavy constant mu*	GVALHRPDVYLLPPAREQLNLR	*Homo sapiens, Pongo abelii*	323	344	207.22	2
**22**	*Q5REL2 Annexin A1*	AAYLQETGKPLDETLK	Boreoeutheria	82	97	106.38	2
		AAYLQETGKPLDETLKK	Boreoeutheria	82	98	150.59	3
**23**	*P08246 Neutrophil elastase*	RLGNGVQCLAMGWGLLGR	*Homo sapiens*	144	161	149.22	3
**24**	*P08311 Cathepsin G*	AQEGLRPGTLCTV	*Homo sapiens*	132	144	167.11	2
		AQEGLRPGTLCTVAGWGR	*Homo sapiens*	132	149	208.17	2
		AAFKGDSGGPLLCNNVAHGIVSYGK	*Homo sapiens*	195	219	311.95	2
**25**	*P01591 Immunoglobulin J chain*	CYTAVVPLVYGGETK	Hominoidea	131	145	206.1	2
		MVETALTPDACYPD	Hominoidea	146	159	204.07	1

Table S2-*Continued.*

**Protein ID**	**Leading Razor Protein**	**Peptide**	***Species***	**Start position**	**End position**	**Score**	**No. of MS2**
**26**	*P11684 Uteroglobin*	VIETLLMDTPSSYEAAMELF	*Homo sapiens*, *Pan* sp.	30	49	211.46	4
		VIETLLMDTPSSYEAAMELFSPD	*Homo sapiens*, *Pan* sp.	30	52	187.44	2
		KLVDTLPQKPRESIIK	*Homo sapiens*, *Pan* sp.	64	79	191.93	4
		LVDTLPQKPRESIIK	*Homo sapiens*, *Pan* sp.	65	79	157.68	4
**27**	*P84049 Histone H4*	*aspecific*					
**28**	*P05109 Protein S100-A8*	YSLIKGNFHAVYRDDLKK	Homininae	19	36	161.35	3
		GNFHAVYRDDLKK	Homininae	24	36	189.23	5
**29**	*Q9Y6R7 IgGFc-binding protein*	VVAEVQICHGK	Homininae	5277	5287	138.1	3

Table S2-*Continued.*

Protein ID	Leading Razor Protein	Peptide	*Species*	Start position	End position	Score	No. of MS2
**30**	*P25311 Zinc-alpha-2-glycoprotein*	HVEDVPAFQALGSLNDLQFFR	Homininae	40	60	332.61	2
		HVEDVPAFQALGSLNDLQFFRYNSK	Homininae	40	64	208.17	1
		EIPAWVPFDPAAQITK	Homininae	150	165	123.75	1
**31**	*P10854 Histone H2B type 1-M*	*aspecific*					
**32**	*P52480 Pyruvate kinase PKM*	*aspecific*					
**33**	*Q6FI13 Histone H2A type 2-A*	*aspecific*					
**34**	*P20160 Azurocidin*	PGVSTVVLGAYDLRR	*Homo sapiens*	74	88	176.86	1
**35**	*Q5R6Y1 Alpha-enolase*	AAVPSGASTGIYEALELR	*Homo sapiens* + several bacteria	33	50	150.16	2
		LAMQEFMILPVGAANFR	Bilateria	163	179	207.47	4

Table S2-*Continued.*

**Protein ID**	**Leading Razor Protein**	**Peptide**	***Species***	**Start position**	**End position**	**Score**	**No. of MS2**
**36**	*Q9U281 Histone H3.3 type 2*	*aspecific*					
**37**	*P01024 Complement C3*	TMQALPYSTVGNSNNYLHLSVLR	*Homo sapiens*	440	462	191.5	1
**38**	*P09228 Cystatin-SA*	SPQEEDRIIEGGIYDADLNDER	*Homo sapiens*	22	43	215.68	1
		IIEGGIYDADLNDERVQR	*Homo sapiens*	29	46	244.08	4
		ALHFVISEYNKATEDEYYRR	*Homo sapiens*	47	66	289.32	2
**39**	*Q9NP55 BPI fold-containing family A member 1*	VLPELVQGNVCPLVNEVLR	*Homo sapiens*	214	232	300.51	5
**40**	*P29401 Transketolase*	SVPTSTVFYPSDGVATEK	Catarrhini	439	456	137.4	1
**41**	*P84198 Vimentin*	SLYASSPGGVYATR	Amniota	51	64	164.48	1
		ILLAELEQLKGQGK	Amniota	130	143	148.68	3

Table S2-*Continued.*

Protein ID	Leading Razor Protein	Peptide	*Species*	Start position	End position	Score	No. of MS2
**42**	*P23280 Carbonic anhydrase 6*	TTLTGLDVQDMLPR	*Homo sapiens*	194	207	257.09	8
		TLTGLDVQDMLPR	*Homo sapiens*	195	207	136.02	1
**43**	*P80188 Neutrophil gelatinase-associated lipocalin*	TFVPGCQPGEFTLGNIK	*Homo sapiens*	102	118	166.45	3
**44**	*P01780 Immunoglobulin heavy variable 3-7*	*aspecific*					
**45**	*P04350 Tubulin beta-4A chain*	*aspecific*					
**46**	*P01861 Immunoglobulin heavy constant gamma 4*	*aspecific*					
**47**	*P10719 ATP synthase subunit beta, mitochondrial*	*aspecific*					

Table S2-*Continued.*

**Protein ID**	**Leading Razor Protein**	**Peptide**	***Species***	**Start position**	**End position**	**Score**	**No. of MS2**
**48**	*P30740 Leukocyte elastase inhibito*	TYNFLPEFLVSTQK	*Homo sapiens*	97	110	129.71	1
**49**	*Q96DA0 Zymogen granule protein 16 homolog B*	GMVMYTSKDRYFYFGK	*Homo sapiens*	132	147	112.17	1
**50**	*P01023 Alpha-2-macroglobulin*	LLIYAVLPTGDVIGDSAK	Catarrhini	540	557	171.29	2
		VDLSFSPSQSLPASHAHLR	Catarrhini	568	586	85.203	1
**51**	*O57672 Glyceraldehyde-3-phosphate dehydrogenase*	*aspecific*					
**52**	*P68361 Tubulin alpha-1B chain*	*aspecific*					
**53**	*Q3SX09 Hemoglobin fetal subunit beta*	RFGSEFSPELQASFQK	Bovinae	172	187	136.63	1
**54**	*P02769 Albumin **	RHPEYAVSVLLR	Pecora	360	371	179.8	1

Table S2-*Continued.*

**Protein ID**	**Leading Razor Protein**	**Peptide**	***Species***	**Start position**	**End position**	**Score**	**No. of MS2**
**55**	*Q9Z1P2 Alpha-actinin-1*	*aspecific*					
**56**	*P02662 Alpha-S1-casein*	PIGSENSEK	*Bos taurus, Bubalus bubalis* **	185	193	74.464	1
**57**	*P01966 Hemoglobin subunit alpha*	VGGHAAEYGAEALER	Bovinae	18	32	186.69	3
**58**	*P02787 Serotransferrin*	HSTIFENLANK	Simiiformes	226	236	303.22	1
**59**	*Q76LA0 Cystatin-B*	VHVGDEDFVHLR	Catarrhini, *Dryobates pubescens*	57	68	222.28	5
		VFQSLPHENKPLTLSNYQTNKAK	Simiiformes	69	91	100.79	1
**60**	*P17990 Gamma-hordein-1*	EFLLQQCRPV	*Hordeum vulgare, Triticum aestivum*	168	177	163.67	1

Table S2-*Continued.*

**Protein ID**	**Leading Razor Protein**	**Peptide**	***Species***	**Start position**	**End position**	**Score**	**No. of MS2**
		VMQQQCCLQLAQIPEQYK	*Hordeum vulgare, Triticum aestivum*	195	212	108.18	1
		CTAIDSIVHAIFMQQGQR	*Hordeum vulgare, Triticum aestivum*	213	230	128.45	1
		APFVGVVTGVGGQ	*Hordeum vulgare, Triticum aestivum*	293	305	156.35	1
**61**	*Q92176 Coronin-1A*	YFEITSEAPFLHYLSMFSSK	Eutheria	294	313	103.26	1
		RLDRLEETVQAK	Eutheria	450	461	194.86	2
**62**	*ENSBTAP00000038253 Keratin type II cytoskeletal 1*	TYSLEPLFEAYISR	*Bos* sp., *Bison bison bison*	220	233	212.09	2
**63**	*Q3ZBD7 Glucose-6-phosphate isomerase*	*aspecific*					

Table S2-*Continued.*

Protein ID	Leading Razor Protein	Peptide	*Species*	Start position	End position	Score	No. of MS2
**64**	*P01700 Immunoglobulin lambda variable 1-47*	LLIYSNNQRPSGVPDRFSGSK	*Homo sapiens, Mus musculus*	66	86	124.31	3
**65**	*P04075 Fructose-bisphosphate aldolase A*	*aspecific*					
**66**	*P19181 Ig heavy chain V region 5A*	*aspecific*					
**67**	*P33048 Beta-casein*	DMPIQAFLLYQEPVLGPVR	Bovidae **	197	215	146.81	1
		LYQEPVLGPVRGPFPILV	Bovidae **	205	222	182.02	1
**68**	*P02070 Hemoglobin subunit beta*	VVAGVANALAHR	Artiodactyla	132	143	104.79	1
		VVAGVANALAHRYH	Artiodactyla	132	145	142.08	2
**69**	*Q3SZR3 Alpha-1-acid glycoprotein*	AIQAAFFYLEPR	*Bos* sp., *Rupicapra rupicapra*	62	73	121.9	1

Table S2-*Continued.*

**Protein ID**	**Leading Razor Protein**	**Peptide**	***Species***	**Start position**	**End position**	**Score**	**No. of MS2**
**70**	*P0CN30 Elongation factor 1-alpha*	*aspecific*					
**71**	*Q5R5A3 Antithrombin-III*	EVPLNTIIFMGR	Primates	446	457	138.08	2
**72**	*Q6P698 Plastin-2*	*aspecific*					

* **-** It should be noted that this is a possible lab contamination, however this protein was not found in the blank. While it should be observed with caution, this is assumed to be authentic, also supported by the presence of other bovine proteins.
** **-** Several bovine milk proteins have exact replicas in the NCBI database with Staphylococcaceae, especially Jeotgalicoccus sp.. These are observed with caution, but it is unlikely that these are correct (likelihood of at least 3 exactly the same proteins is low), and are more likely some form of genome contamination for these species.

Table S3- UV2 protein summary.

**Protein no**	**Leading Razor Protein**	**Species Specificity of Peptides Recovered**	**No. of Identified Razor+ Unique Peptides (semi-tryptic)**	**% Coverage (not razor)**	**No. of MS2 spectra (razor)**
**1**	P02453 Collagen alpha-1(I) chain	Bovinae	311	54.7	467
**2**	P02465 Collagen alpha-2(I) chain	Bovinae	249	50.9	345
**3**	P04258 Collagen alpha-1(III) chain	*Bos* sp., *Bison bison bison*	118	37.2	186
**4**	Q3SX09 Hemoglobin fetal subunit beta	*Bos* sp., *Bison bison bison*	17	50.7	21
**5**	P02768 Albumin	Hominoidea	9	14.9	10
**6**	ENSBTAP00000038253 Keratin type II cytoskeletal 1	*Bos* sp., *Bison bison bison*	8	34.7	9
**7**	Q25010 Actin, cytoplasmic A3a	aspecific	7	14.6	8
**8**	P02788 Lactotransferrin	*Homo sapiens*	6	12	6
**9**	P84049 Histone H4	aspecific	6	46.6	8
**10**	P02769 Albumin *	Bovinae	6	12.5	8
**11**	P02662 Alpha-S1-casein *	Bovinae **	6	22.1	7
**12**	P0DUB6 Alpha-amylase 1A	Eutheria	5	10.2	7
**13**	P10854 Histone H2B type 1-M	aspecific	5	19.8	5
**14**	Q6FI13 Histone H2A type 2-A	aspecific	5	35.4	8

Table S3- *Continued.*

Protein no	Leading Razor Protein	Species Specificity of Peptides Recovered	No. of Identified Razor+ Unique Peptides (semi-tryptic)	% Coverage (not razor)	No. of MS2 spectra (razor)
**15**	P01036 Cystatin-S	*Homo sapiens*	4	21.3	4
**16**	P31025 Lipocalin-1	Hominoidea	3	17	5
**17**	P01966 Hemoglobin subunit alpha	Bovinae	3	25.4	5
**18**	P02070 Hemoglobin subunit beta	Artiodactyla	3	42.1	3
**19**	Q9HC84 Mucin-5B	Catarrhini	2	1.5	2
**20**	P01833 Polymeric immunoglobulin receptor	*Homo sapiens*	2	3.9	3
**21**	P01834 Immunoglobulin kappa constant	aspecific	2	30.8	3
**22**	P25311 Zinc-alpha-2-glycoprotein	Homininae	2	11.4	3
**23**	Q5XQN5 Keratin, type II cytoskeletal 5	Pecora	2	34.8	2

* - It should be noted that this is a possible lab contamination, however this protein was not found in the blank. While it should be observed with caution, this is assumed to be authentic, also supported by the presence of other bovine proteins.

** - Several bovine milk proteins have exact replicas in the NCBI database with Staphylococcaceae, especially Jeotgalicoccus sp.. These are observed with caution, but it is unlikely that these are correct (likelyhood of at least 3 exactly the same proteins is low), and are more likely some form of genome contamination for these species.

Table S4- UV2 specific peptides.

**Protein ID**	***Leading Razor Protein***	**Peptide**	**Species**	**Start position**	**End position**	**Score**	**No. of MS2**
1	P02453 Collagen alpha-1(I) chain	PGAVGAKGEGGPQGPR	Bovinae	345	360	102.65	2
		AVGAKGEGGPQGPR	Bovinae	347	360	105.03	1
		GEGGPQGPRGSEGPQGVR	Bovinae	352	369	100.35	1
		GGPQGPRGSEGPQGVR	Bovinae	354	369	79.317	1
2	P02465 Collagen alpha-2(I) chain	GPSGDPGKAGEKGHAGLAGAR	Bovinae	497	517	172.84	2
		SGETGASGPPGFVGEKGPSG	Bovinae	829	848	174.24	1
		SGETGASGPPGFVGEKGPSGEPG	Bovinae	829	851	134.14	1
		PGNAGPVGAAGAPGPQGPVGPVGK	Bovinae	949	972	195.46	1
		GNAGPVGAAGAPGPQGPVGPVGK	Bovinae	950	972	87.208	1
		NAGPVGAAGAPGPQGPVGPVGK	Bovinae	951	972	95.5	1

Table S4- *Continued*.

**Protein ID**	***Leading Razor Protein***	**Peptide**	**Species**	**Start position**	**End position**	**Score**	**No. of MS2**
2	P02465 Collagen alpha-2(I) chain	AGPVGAAGAPGPQGPVGPVGK	Bovinae	952	972	182.15	2
		AGPVGAAGAPGPQGPVGPVGKHGNR	Bovinae	952	976	87.772	1
		GPVGAAGAPGPQGPVGPVGK	Bovinae	953	972	245.94	2
		PVGAAGAPGPQGPVGPVGK	Bovinae	954	972	208.74	2
		AGAPGPQGPVGPVGK	Bovinae	958	972	120.22	1
		GAPGPQGPVGPVGK	Bovinae	959	972	81.263	1
		APGPQGPVGPVGK	Bovinae	960	972	186.92	1
		PGPQGPVGPVGK	Bovinae	961	972	243.33	2
		GPAGPSGPAGKDGRIGQPGAVG	Bovinae	1052	1073	99.569	2
		DGRIGQPGAVGPAGIR	Bovinae	1063	1078	126.07	3

Table S4- ***Continued*.**

**Protein ID**	***Leading Razor Protein***	**Peptide**	**Species**	**Start position**	**End position**	**Score**	**No. of MS2**
2	P02465 Collagen alpha-2(I) chain	GRIGQPGAVGPAGIR	Bovinae	1064	1078	115.24	2
		RIGQPGAVGPAGIR	Bovinae	1065	1078	116.71	2
3	P04258 Collagen alpha-1(III) chain	GESGAPGVPGIAGPR	Bovinae	780	794	79.659	1
		GAPGSPGISGPKGDSGPPGER	*Bos* sp., *Bison bison bison*	912	932	76.027	1
4	Q3SX09 Hemoglobin fetal subunit beta	FFESFGDLSSADAILGNPK	*Bos* sp., *Bison bison bison*	96	114	180.28	1
5	P02768 Albumin	LVNEVTEFAK	Eutheria	66	75	138.06	1
		RHPYFYAPELLFFAK	Hominoidea + *Nocardioides* sp. OK12	169	183	67.136	1
		KLVAASQAALGL	Hominoidea + *Nocardioides* sp. OK13	598	609	103.55	1

Table S4- ***Continued*****.**

**Protein ID**	***Leading Razor Protein***	**Peptide**	**Species**	**Start position**	**End position**	**Score**	**No. of MS2**
6	ENSBTAP00000038253 Keratin type II cytoskeletal 1	TYSLEPLFEAYISR	*Bos* sp., *Bison bison bison*	220	233	199.7	2
		SLEPLFEAYISR	*Bos* sp., *Bison bison bison*	222	233	119.53	1
		FDNLLQEIDFYK	*Bos* sp., *Bison bison bison*	301	312	152.7	1
		RGGSGGGGGSSGGSFISSGGR	*Bos* sp., *Bison bison bison*	561	581	116.45	1
7	Q25010 Actin, cytoplasmic A3a	*aspecific*					
8	P02788 Lactotransferrin	YLGPQYVAGITNLKK	*Homo sapiens*	681	695	74.783	1
9	P84049 Histone H4	*aspecific*					
10	P02769 Albumin *	DAFLGSFLYEYSR	Bovinae	347	359	97.452	1

Table S4- *Continued*.

Protein ID	*Leading Razor Protein*	Peptide	Species	Start position	End position	Score	No. of MS2
11	P02662 Alpha-S1-casein *	FFVAPFPEVF	Bovinae **	23	32	100.07	1
		FFVAPFPEVFGK	Bovinae **	23	34	108.67	1
		FFVAPFPEVFGKEK	Bovinae **	23	36	83.499	1
		YLGYLEQLLR	Bovinae **	91	100	227.3	2
		QELAYFYPELFR	Bovinae **	140	151	77.64	1
12	P0DUB6 Alpha-amylase 1A	AHFSISNSAEDPFIAIHAESKL	Eutheria	490	511	231.37	3
13	P10854 Histone H2B type 1-M	*aspecific*					
14	Q6FI13 Histone H2A type 2-A	*aspecific*					
15	P01036 Cystatin-S	AREQTFGGVNYFFDVEVGR	*Homo sapiens*	73	91	118.46	19
16	P31025 Lipocalin-1	VTMLISGR	Hominoidea	71	78	109.36	2

Table S4- *Continued*.

Protein ID	*Leading Razor Protein*	Peptide	Species	Start position	End position	Score	No. of MS2
17	P01966 Hemoglobin subunit alpha	VGGHAAEYGAEALER	Bovinae	18	32	186.69	3
18	P02070 Hemoglobin subunit beta	VVAGVANALAHR	Artiodactyla	132	143	120.06	1
		VVAGVANALAHRYH	Artiodactyla	132	145	92.925	1
19	Q9HC84 Mucin-5B	LFVESYELILQEGTFK	Simiiformes	957	972	184.1	1
		ELGQVVECSLDFGLVCR	Catarrhini + *Stutzerimonas stutzeri*	2372	2388	107.98	1
20	P01833 Polymeric immunoglobulin receptor	LDIQGTGQLLFSVVINQLR	*Homo sapiens*	193	211	308.84	2
		LVSLTLNLVTR	*Homo sapiens*	526	536	115.78	1
21	P01834 Immunoglobulin kappa constant	*aspecific*					

Table S4- *Continued*.

Protein ID	Leading Razor Protein	Peptide	Species	Start position	End position	Score	No. of MS2
22	P25311 Zinc-alpha-2-glycoprotein	HVEDVPAFQALGSLNDLQFFR	Homininae	40	60	141.97	2
23	Q5XQN5 Keratin, type II cytoskeletal 5	VSLGGAYGAGGFGSR	Pecora	49	63	177.04	1

* - It should be noted that this is a possible lab contamination, however this protein was not found in the blank. While it should be observed with caution, this is assumed to be authentic, also supported by the presence of other bovine proteins.

** - Several bovine milk proteins have exact replicas in the NCBI database with Staphylococcaceae, especially Jeotgalicoccus sp.. These are observed with caution, but it is unlikely that these are correct (likelihood of at least 3 exactly the same proteins is low), and are more likely some form of genome contamination for these species.

Table S5- The nLC column carry-over of Collagen 1 peptides of an archaeological cow bone demonstrated by the number of Peptide-Spectrum Matches (PSMs) identified by MaxQuant for the subsequent two wash injections and the following Uvdal laboratory extraction blank.

Protein	Peptide	PSMs Wash 1	PSMs Wash 2	PSMs Laboratory Blank
COL1A1	DGEAGAQGPPGPAGPAGER	10	1	1
	DGLNGLPGPIGPPGPR	14	5	
	GAAGLPGPK	1		
	GANGAPGIAGAPGFPGAR	3	1	
	GAPGADGPAGAPGTPGPQGIAGQR	14	3	1
	GAPGDRGEPGPPGPAGFAGPPGADGQPGAK	1	1	
	GDAGPPGPAGPAGPPGPIGNVGAPGPK	9	3	
	GDRGETGPAGPPGAPGAPGAPGPVGPAGK	2		
	GDTGAKGEPGPTGIQGPPGPAGEEGKR	1		
	GEAGPSGPAGPTGAR	1		
	GEPGPAGLPGPPGER	1	1	
	GEPGPPGPAGAAGPAGNPGADGQPGAK	2		
	GEPGPPGPAGFAGPPGADGQPGAK	6		1
	GEPGPTGIQGPPGPAGEEGK	2		
	GEPGPTGIQGPPGPAGEEGKR	2	2	2
	GETGPAGPAGPIGPVGAR	2	2	1

Table S5- ***Continued*****.**

Protein	Peptide	PSMs Wash 1	PSMs Wash 2	PSMs Laboratory Blank
COL1A1	GETGPAGPPGAPGAPGAPGPVGPAGK	5		
	GETGPAGRPGEVGPPGPPGPAGEK	3	1	1
	GFPGADGVAGPK	1	1	1
	GFPGLPGPSGEPGK	3	2	
	GFSGLQGPPGPPGSPGEQGPSGASGPAGPR	6	1	
	GLTGPIGPPGPAGAPGDK	2	1	
	GLTGPIGPPGPAGAPGDKGEAGPSGPAGPTGAR	4	2	
	GLTGSPGSPGPDGK	1		
	GNDGATGAAGPPGPTGPAGPPGFPGAVGAK	9	2	
	GPAGPQGPR	1		
	GPPGPMGPPGLAGPPGESGR	9	2	
	GPPGPPGKNGDDGEAGKPGR	2		
	GPPGSAGSPGKDGLNGLPGPIGPPGPR	1		
	GPSGPQGPSGPPGPK	2		1
	GQAGVMGFPGPK	4	1	
	GRPGAPGPAGAR	1		
	GSAGPPGATGFPGAAGR	1	1	1

Table S5- ***Continued*****.**

Protein	Peptide	PSMs Wash 1	PSMs Wash 2	PSMs Laboratory Blank
COL1A1	GVPGPPGAVGPAGK	3		
	GVPGPPGAVGPAGKDGEAGAQGPPGPAGPAGER	1		
	GVQGPPGPAGPR	2		
	GVVGLPGQR	2	1	
	NGDDGEAGKPGRPGER	2	1	
	PGEVGPPGPPGPAGEK	2		
	SGDRGETGPAGPAGPIGPVGAR	7	1	1
	TGPPGPAGQDGRPGPPGPPGAR	1	1	1
	VGPPGPSGNAGPPGPPGPAGK	2		1
COL1A2	EGPVGLPGIDGR	4	2	
	EGPVGLPGIDGRPGPIGPAGAR	2	1	
	GAAGLPGVAGAPGLPGPR	2	1	
	GAPGAIGAPGPAGANGDR	1		
	GAPGAIGAPGPAGANGDRGEAGPAGPAGPAGPR	3	1	
	GAPGPDGNNGAQGPPGLQGVQGGK	1		
	GDGGPPGATGFPGAAGR	2		1
	GEAGPAGPAGPAGPR	1		

Table S5- *Continued*.

Protein	Peptide	PSMs Wash 1	PSMs Wash 2	PSMs Laboratory Blank
COL1A2	GELGPVGNPGPAGPAGPR	5	1	
	GENGPVGPTGPVGAAGPSGPNGPPGPAGSR	2	1	
	GEPGAVGQPGPPGPSGEEGKR	1		
	GEPGNIGFPGPK	3	1	
	GEPGPAGAVGPAGAVGPR	2	1	
	GEPGVVGAPGTAGPSGPSGLPGER	2		
	GEQGPAGPPGFQGLPGPAGTAGEAGKPGER	2	1	
	GESGNKGEPGAVGQPGPPGPSGEEGK	1		
	GESGNKGEPGAVGQPGPPGPSGEEGKR	3	1	
	GEVGLPGLSGPVGPPGNPGANGLPGAK	1	1	
	GEVGPAGPNGFAGPAGAAGQPGAK	4	1	
	GEVGPAGPNGFAGPAGAAGQPGAKGER	2		
	GFPGSPGNIGPAGK	2	1	1
	GFPGTPGLPGFK	1		
	GHNGLQGLPGLAGHHGDQGAPGAVGPAGPR	2		
	GIPGEFGLPGPAGAR	3	2	
	GIPGPVGAAGATGAR	1		

Table S5- ***Continued*****.**

Protein	Peptide	PSMs Wash 1	PSMs Wash 2	PSMs Laboratory Blank
COL1A2	GLVGEPGPAGSK	1		
	GPAGPSGPAGK	1		
	GPKGENGPVGPTGPVGAAGPSGPNGPPGPAGSR	3	1	
	GPNGDSGRPGEPGLMGPR	2	1	
	GPPGESGAAGPTGPIGSR	3	1	1
	GPPGNVGNPGVNGAPGEAGR	2		
	GPSGEPGTAGPPGTPGPQGLLGAPGFLGLPGSR	3	1	
	GPSGPPGPDGNKGEPGVVGAPGTAGPSGPSGLPGER	4	2	
	GPSGPQGIR	2		
	GSDGSVGPVGPAGPIGSAGPPGFPGAPGPK	2	2	
	GSTGEIGPAGPPGPPGLR	2	1	
	GVVGPQGAR	1		
	GYPGNAGPVGAAGAPGPQGPVGPVGK	1		
	HGNRGEPGPAGAVGPAGAVGPR	2		1
	IGQPGAVGPAGIR	4	2	1
	PGPIGPAGAR	2		
	RGSTGEIGPAGPPGPPGLR	1		

Table S5- *Continued*.

Protein	Peptide	PSMs Wash 1	PSMs Wash 2	PSMs Laboratory Blank
COL1A2	GLPGVAGSVGEPGPLGIAGPPGAR	2	3	
	SGETGASGPPGFVGEK	1	1	1
	TGPPGPSGISGPPGPPGPAGK	4		1
	VGAPGPAGAR	1		

Table S6 - Count of peptides used in the bulk deamination calculation.

Sample	Category	Count	Amino Acid
UV1	Binder	33	N
UV1	Human	367	N
UV1	Oat/Barley	22	N
UV1	Binder	38	Q
UV1	Casein	2	Q
UV1	Human	322	Q
UV1	Oat/Barley	32	Q
UV2	Binder	32	N
UV2	Casein	1	N
UV2	Human	25	N
UV2	Oat/Barley	1	N
UV2	Binder	45	Q
UV2	Human	33	Q
UV2	Oat/Barley	2	Q

Table S7 - Deamidation of binder proteins in both samples, averaged from raw deamiDATE output.

Sample	N Deamidation	N Occurrences	Q Deamidation	Q Occurrences
**UV1**	13.31999053	33	2.284972006	38
**UV2**	13.50648094	32	1.384805112	45

[1] Brademan, D. R., Riley, N. M., Kwiecien, N. W. & Coon, J. J. Interactive Peptide Spectral Annotator: A Versatile Web-based Tool for Proteomic Applications. *Mol. Cell. Proteomics* **18**, S193–S201 (2019).
